# Entering into a self-regulated learning mode prevents detrimental effects of feedback removal on memory

**DOI:** 10.1038/s41539-022-00150-x

**Published:** 2023-01-06

**Authors:** Peter Vavra, Leo Sokolovič, Emanuele Porcu, Pablo Ripollés, Antoni Rodriguez-Fornells, Toemme Noesselt

**Affiliations:** 1 Department of Biological Psychology, Otto-von-Guericke-University Magdeburg, Magdeburg, Germany; 2 Center of Behavioral Brain Sciences, Magdeburg, Germany; 3 Department of Psychology, New York University, New York, NY USA; 4 Music and Audio Research Lab (MARL), New York University, New York, NY USA; 5 Center for Language, Music, and Emotion (CLaME), New York University, Max-Planck Institute, New York, NY USA; 6 Department of Cognition, Development, and Educational Science, Institute of Neuroscience, University of Barcelona, L’Hospitalet de Llobregat, 08097 Barcelona, Spain; 7 Cognition and Brain Plasticity Group, Bellvitge Biomedical Research Institute (IDIBELL), L’Hospitalet de Llobregat, 08097 Barcelona, Spain; 8 Catalan Institution for Research and Advanced Studies, ICREA, Barcelona, Spain

**Keywords:** Human behaviour, Learning and memory

## Abstract

Incentives can decrease performance by undermining intrinsic motivation. How such an interplay of external reinforcers and internal self-regulation influences memory processes, however, is less known. Here, we investigated their interaction on memory performance while learning the meaning of new-words from their context. Specifically, participants inferred congruent meanings of new-words from semantic context (congruent trials) or lack of congruence (incongruent trials), while receiving external feedback in the first or second half of trials only. Removing feedback during learning of congruent word meanings lowered subsequent recognition rates a day later, whereas recognition remained high in the group, which received feedback only in the second half. In contrast, feedback did not substantially alter recognition rates for learning that new-words had no congruent meanings. Our findings suggest that external reinforcers can selectively impair memories if internal self-regulated processes are not already established, but whether they do so depends on what is being learned (specific word-meanings vs. unspecific incongruence). This highlights the relevance of self-regulated learning in education to support stable memory formation.

## Introduction

The interplay of internal motivational states and external incentives governs our behavior^1–4^. Usually, intrinsic motivation refers to those activities that are engaged for their very own sake (e.g., enjoyment and pleasure) and are not necessarily associated with a particular instrumental and external benefit (e.g., incentives)^5^. A key finding^6^, the *undermining effect*, posits that the introduction of extrinsic incentives “crowds out” intrinsic motivation. Moreover, performance plummets when the incentives are subsequently removed. While there has been some controversy about the robustness of this effect^7,8^, a quantitative meta-analysis^5^ found evidence for it: explicit rewards increase task performance, but reduce the influence of intrinsic motivation on the task outcome. A determining factor accounting for previous differences across studies appears to be the use of distinct performance measures (e.g., qualitative vs. quantitative). In educational contexts, however, feedback (a form of reinforcement signal, which can be positive or negative) cannot only be withheld, but is often added after initial unsupervised behavior, yet this sequence has drawn considerably less research so far. Moreover, there is another crucial dimension to consider: motivational factors may not only affect performance during the learning session (e.g., how well one understands the meaning of a new-word when first encountering it), but the interplay between different motivational factors might also influence longer-lasting memory processes (e.g., remembering the new vocabulary and reducing natural forgetting).

Learning the meaning of new words from context provides an ecologically valid paradigm to study how new memory traces are established. In our previous work, participants learned the meaning of new-words from a semantic context formed by two sentences^9–11^. The meanings in the two sentences were congruent for half of the new-words and, thus, the second sentence disambiguated between different meanings (e.g., *“Every Sunday the grandmothers went to the jedin”* and *“The man was buried at the jedin”* (likely congruent meaning: graveyard)); for the other half of the new-words, the sentences were incongruent (i.e., no coherent meaning could be derived, e.g., *“Every night the astronomer watched the heutil”* (likely meaning: moon, stars or some other extraterrestrial object) and “*Every morning the co-workers drink heutil*” (likely meaning: coffee, tea)). Participants were told to extract the meaning of the new-word when the meaning was congruent and to detect the incongruence otherwise. Critically, we showed that, in absence of any explicit reward, successful learning was tightly coupled with increased self-reports of pleasure, enhanced electrodermal activity and, most importantly, heightened fMRI activity in the dopaminergic (DA) loop^9,10^ mainly formed by the substantia nigra/ventral tegmental area complex (SN/VTA), the hippocampus (HP) and the ventral striatum (VS; hereafter referred to as SN/VTA-HP loop^12–15^. Moreover, intrinsically generated reward-related signals paralleled an increase in memory retention for these new-words in a surprise memory-test assessing long-term memory on the next day and was still detected when testing recognition memory after 7 days^10^. The successful discovery of the incongruence between the meanings evoked by the two sentences was neither related to a heightened behavioral or physiological measure, nor to increased reward-related activity in the SN/VTA-HP loop. In our subsequent work^11^, we employed a double-blind, within-subject randomized pharmacological manipulation of the dopaminergic system using l-DOPA and risperidone, showing converging evidence. Taken together, it seems that dopaminergic signaling improved performance during the initial learning phase, as well as long-term memory for the new-words learned from congruent contexts (i.e., DA-dependent motivated-learning) while we did not observe a significant relationship for incongruent contexts (i.e., DA-independent learning).

Learning is often driven by reinforcement. Neurobiologically, there is accumulating evidence showing that external rewards can boost learning and memory performance by activating the SN/VTA-loop and ultimately enhance long-term memory storage (though note that other neuromodulators might also be involved^16^). In addition to external primary and secondary reinforcers like food or money, external performance feedback can also boost activity in SN/VTA and striatum^17,18^, as well as modulate long-term memory^19^. Importantly for our work, this SN/VTA-HP loop can also be modulated in absence of any external signal, e.g., by intrinsic states like curiosity^20,21^, or by self-regulated learning^9,10^.

Given that both extrinsic feedback and intrinsic learning signals appear to be DA-dependent and apparently activate at least parts of the same reward-related SN/VTA-HP loop, a crucial question is how these two processes interact or to which extent they compete^6,22–24^ and whether the timing at which feedback is introduced or withdrawn further impacts the interplay. However, there is still an ongoing debate to what extent feedback boosts^25,26^ or even suppresses^27–29^ longer-lasting memories. For instance, some studies have reported enhanced memory performance as a function of testing (the so-called testing effects^30^), with feedback further boosting the beneficial effects of testing. In other contexts however, feedback might come at a cost and may reduce later recall, e.g., of word associations^31^, while others have suggested that feedback may not affect word learning, especially if performance feedback is used^32,33^. Together, the effects of feedback are still poorly understood especially in the field of word learning^26^.

Here, we extend our prior research by adding trial-based performance feedback on participants’ accuracy in our new-word learning paradigm^9–11^. External performance feedback could provide a more ecologically valid setting for learning paradigms in humans than presenting primary or secondary reinforcers and might interact with internally triggered processes in a complex manner. Our aim was to investigate how two distinct processes—external feedback and intrinsic self-regulated learning—interact. Crucially, we compared learning trials: congruent trials previously related to intrinsically triggered dopaminergic release (discovering a meaning from a congruent context) with trials where such an intrinsically triggered DA-signal seems less pronounced (incongruent contexts, DA-independent). Hence, the inclusion of both congruent and incongruent trials allows us to study the effect of feedback on two different learning processes using well-matched stimuli within the same paradigm, which in turn seem to rely to different degrees on DA transmission. We hypothesized that immediate meaning extraction and later memory performance should be differentially affected by adding external feedback. Importantly, memory-related effects should differ depending on the two trial-based congruency in our experiment. During congruent trials, an interplay of DA-dependent external feedback with DA-dependent intrinsic signals should be observed since dopaminergic signaling is related to learning performance and memory consolidation for this condition^11^. In contrast, during incongruent trials the task is to learn that the meanings between the two sentences were incongruent and to store this more general association in memory (i.e., that a particular new-word has an incongruent meaning). Further, we hypothesized that the timing is critical at which external feedback is introduced. According to the undermining effect, feedback that was presented from the beginning of the task should decrease performance once it is removed. Although not implied by the undermining effect, adding feedback after an initial feedback-free phase might also increase performance. We tested these hypotheses in two different settings: a laboratory experiment and an online experiment using two independent samples.

## Results

### Experiment 1 (laboratory)

**Fig. 1:**
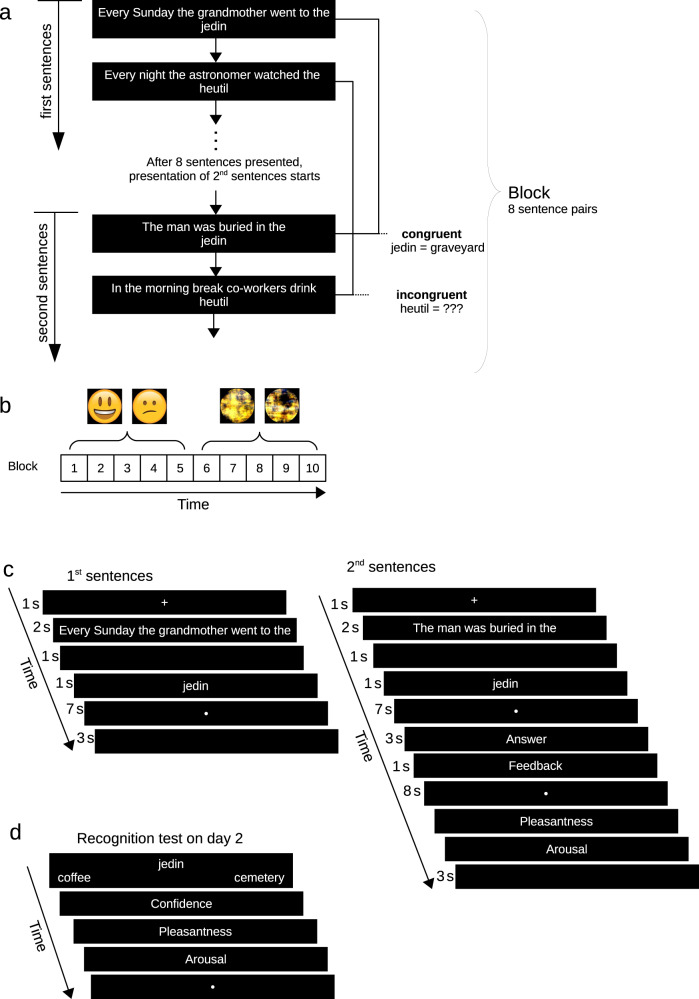
Experimental Procedure for experiments 1 and 2. **a** Example of a block with 8 first and 8 second sentences. In each block 8 pairs of sentences were presented. In the first half of each block all eight first sentences were presented, all ending in a different new-word (see above for one congruent and incongruent example sentence pair each). After the presentation of all 8 first sentences, the second sentences for those same new-words were shown in a randomized order relative to the first sentences. **b** Exemplary overall block structure of the experiment for one participant who started with feedback (blocks 1–5) and switched to no feedback in the second half (block 6–10). Across participants feedback order was counter-balanced, i.e., feedback was present in the first or second half of the blocks (feedback first or no feedback first). All participants received feedback in form of a smiley or frownie and scrambled smileys/frownies (no feedback). **c** An exemplary congruent trial in the learning session adapted from Ripollés et al.^10^. In each paired learning trial, two sentences ending in the same new-word were presented. For congruent trials, the two sentences evoke the same congruent meaning (“graveyard”) and thus DA-dependent word learning can occur (i.e., *jedin* means “graveyard”). Incongruent trials follow the same structure with the difference that the two sentences do not evoke the same meaning (i.e., participants can only learn that this new-word had an two incongruent meanings, DA-independent). After the presentation of the new word, subject’s response and feedback, subjective ratings of pleasantness and arousal were obtained. **d** An example of a recognition test trial on day 2. All new-words (both congruent and incongruent) from the learning session were presented in a randomized order with two possible meanings to choose from. In addition, participants could indicate that the meaning for that new-word had been incongruent, or that they didn’t know. After indicating their answer, participants again rated their pleasantness and arousal, as well as confidence.

Sixty healthy participants completed an altered version of our word-learning task (see also *Methods* and Fig. 1) in the laboratory. In this task, participants read sentences, which end in a new-word. Importantly, each new-word was presented at the end of two sentences and these pairs of sentences could be congruent (congruent trials; e.g., “Every Sunday the grandmother went to the *jedin*” (first sentence); “The man was buried at the *jedin*” (second sentence); congruent meaning: graveyard), allowing the inference of a coherent meaning of the new-word; or sentences could be incongruent (incongruent trials; e.g., “I bought tickets for the *jardy*”; “The workers drink coffee with *jardy*”; first meaning: concert/football game etc., second meaning: milk, sugar, alcohol), such that no unique meaning fits both sentences. Sentences were presented in blocks of 8 sentence pairs, such that 8 first sentences and the associated new-words were presented first (opening all 8 sentence pairs) before reading the corresponding second sentences (closing the sentence pairs). Immediately after reading each second sentence and the associated new-word, participants verbally responded by either saying what the congruent meaning of the new-word had been, or indicating that the sentences had been incongruent. Finally, participants could also indicate that they were unsure by remaining silent or by saying “‘I don’t know’”. In contrast to our previous work, in the current version of the task we provided participants with trial-based informative performance feedback on half the trials (smileys, frownies) or showed them non-informative placeholders pictures on the other half (scrambled smileys/frownies; here-after called “no feedback”). After receiving feedback (or seeing scrambled placeholders, see Fig. 1b), participants then rated their subjective states on pleasantness and arousal scales, before proceeding to read the next second sentence (see Fig. 1c right side). Moreover, participants were divided into two groups, one in which feedback was provided in the first half of the trials and then removed (feedback first) and one in which feedback was added in the second half of the trials (no-feedback first).

Together, this resulted in a two (congruence: congruent/incongruent) by two (feedback: feedback/no-feedback) by two (order: feedback first/no-feedback first) design, with the factors congruence and feedback being within-subject and the factor order between-subject. Responses during the learning session were counted as correct only if participants either provided the correct word-meaning on congruent trials, or correctly indicated that the two meanings were inconsistent on incongruent trials.

After ~24 h, participants completed a surprise memory test to assess their learning: Thirty-one participants completed a free-recall memory test where they had to indicate which new-words they remembered and indicate their meaning, or that the two sentences had been incongruent. The other twenty-nine participants completed a recognition test (chance level was 25%; see *Methods* for details).

During the learning and recognition session participants also rated pleasantness, arousal and confidence after each trial, in order to assess the subjective states underlying the behavioral responses (see Fig. 1, see Supplementary Materials for Results and Discussion).

### Adding external feedback improves immediate performance of meaning extraction on day 1

**Fig. 2:**
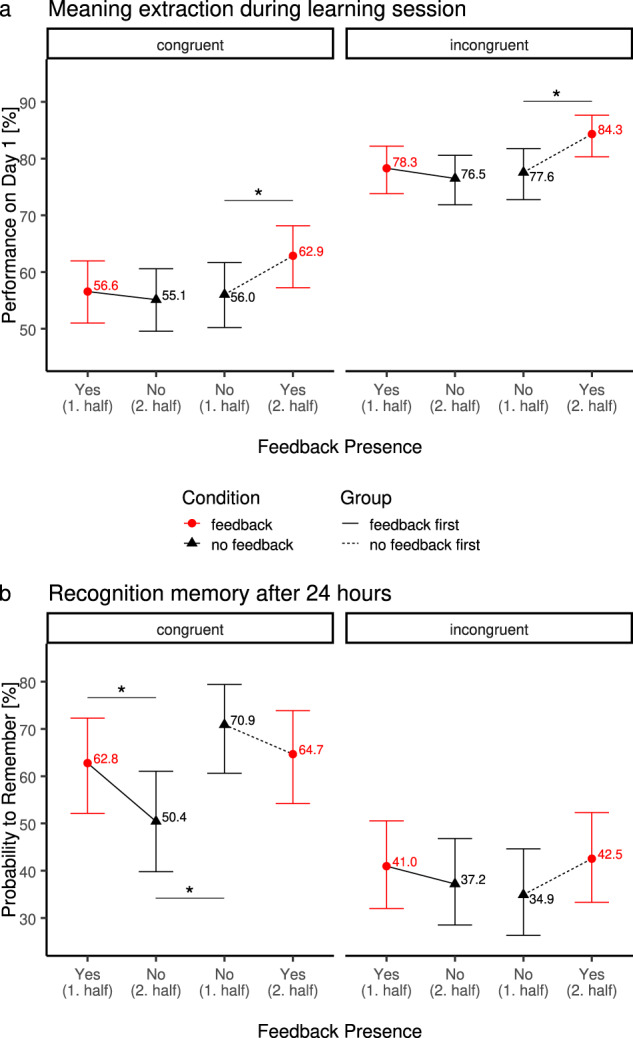
Behavioural results of Experiment 1 (laboratory). Performance on new-word meaning extraction task on day 1 (**a**) and recognition memory on day 2 (**b**) in Experiment 1 (laboratory). Trials with no congruent meaning (incongruent) are displayed on the right, trials with congruent meaning (congruent) on the left, separately for feedback order (between subject effect; lines connect data points for the feedback first and no feedback first groups; full line: started with feedback; dotted line: started without feedback) and feedback presence (within-subject effect; red dot: feedback; black triangle: no feedback). Note that feedback presence is plotted in order of occurrence on *x*-axis (i.e., reversed feedback presence for the two groups). Lines between data points indicate identical within-group measures differing only in feedback presence. Error bars indicate 95% confidence interval. **a** Immediate performance during meaning extraction task (implicit learning). Across conditions incongruent trials (right side) showed a higher level of performance than congruent trials. Informative feedback improved performance for the group of participants who started off without feedback and started receiving feedback on their answers in the second half of the experiment. Importantly, this pattern was observed for both congruent and incongruent trials (*p* < 0.023 and *p* < 0.004 for congruent and incongruent trials, respectively). **b** Recognition performance on day 2 as a function of congruence of meaning across sentences, feedback order and feedback presence (chance level = 25%). In contrast to day 1, incongruent new-words were less likely to be remembered as having had incongruent meanings across both sentences than the specific meaning of congruent new-words. For incongruent trials, there was a trend for feedback boosting recollection rates. For congruent trials, removing feedback in the second half of the experiment lead to a substantial drop in recognition rates a day later (*p* < 0.034), whereas no effect was found if feedback was added in the second half of trials (i.e., if participants started the learning phase with no-feedback, *p* > 0.24).

We first assessed whether external feedback influenced immediate task performance (Fig. 2a) on day 1 using a generalized linear mixed model with the within-subject factors feedback (feedback/no feedback) and congruence (congruent/incongruent) and the between-subject factor order (feedback first/no feedback first), with a random intercept per subject.

On average, participants (*N* = 60) were able to correctly infer the meaning of new-words from the congruent contextual information (congruent trials: 57.7 ± 1.7% correct, see Fig. 2; this learning rate is in accord with our previous work^9–11^. They were also able to correctly indicate when two sentences were incongruent (incongruent trials: 79.4 ± 1.4% correct; main effect of congruence: *χ*^2^(1) = 248.6, *p* < 0.0001, see Fig. 2a).

Importantly, receiving feedback improved the overall performance of meaning extraction during the implicit learning phase (4.7% difference; main effect of feedback: *χ*^2^(1) = 10.9, *p* = 0.001). However, this effect was mostly driven by the order in which feedback was provided: When participants who began without feedback started receiving feedback in the second half of the experiment, their performance improved substantially (67.7% to 75.1% when collapsing across incongruent and congruent trials, odds-ratio = 0.696 ± 0.069, *p* = 0.0003); the corresponding drop in performance for the group of participants for whom the feedback was withdrawn was substantially smaller (68.4% with feedback to 66.7% without, odds-ratio=0.923 ± 0.083, *p* = 0.37; interaction feedback-by-order: *χ*^2^(1) = 4.42, *p* = 0.04). These effects of feedback were not significantly different for the congruent/incongruent trials (congruence-by-feedback: *χ*^2^(1) = 0.57, *p* = 0.45, congruence-by-order: *χ*^2^(1) = 0.36, *p* = 0.55; congruence-by-feedback-by-order: *χ*^2^(1) = 0.18, *p* = 0.67).

Overall, present results showed that introducing feedback in the second half of the experiment improves immediate performance of meaning extraction and detection of incongruent meanings, whereas the withdrawal of feedback led to no significant drop in performance on day 1 (see Fig. 2a).

### Removing external feedback decreases long-term memory for specific new-word meanings

To assess how this external feedback influenced memory consolidation processes, we next examined performance levels of the surprise memory test 24 h later. About half of the participants performed a free-recall test, while the other half performed a forced-choice recognition test (with 25% chance level, see Materials). Since under free-recall, participants’ performance was very low (22.1% overall and 4.4% on congruent trials) floor effects are likely. In contrast, performances were substantially higher on the recognition test and comparable in size with our previous work^10,11^ using the same task and memory test (congruent: 42.8%; incongruent: 36.5%), thus only the data from the recognition group is analyzed below.

To characterize how the presence of feedback affects recognition memory, we focused our analysis on the subset of new-words, which were correctly answered on day 1, and could, thus, be either remembered or forgotten a day later in accord with our earlier approach^10,11^ (see Fig. 2b), using a generalized linear mixed model with the within-subject factors feedback (feedback/no feedback) and congruence (congruent/incongruent) and the between-subject factor order (feedback first/no feedback first), with a random intercept per subject. Note that by focusing on new-words, which were correctly answered on day 1, all of these new-words were paired either with positive feedback or no feedback. Participants (*N* = 29) were, on average, more likely to remember the congruent new-words (congruent: 62.4% vs. incongruent: 38.9%, main effect of congruence: *χ*^2^(1) = 78.32 *p* < 0.0001) after 24 h. This pattern remained significant when comparing the raw recognition rates of congruent and incongruent trials (i.e., independent of whether they were correctly solved on day 1; *χ*^2^(1) = 9.18, *p* < 0.002) and is hence not due to the smaller number of to-be-remembered congruent items on day 1.

Importantly, the timing of presence of feedback (first or second half) significantly influenced the probability to remember, but differently so for congruent and incongruent trials: For congruent new-words only, withdrawing feedback lead to a significant drop in memory performance (62.8% vs. 50.4%, odds-ratio=1.66 ± 0.4, *p* = 0.034) while the decrease in performance did not significantly differ for the group who started off without feedback (odds-ratio = 0.75 ± 0.18, *p* = 0.24; for congruent only, interaction of feedback by order: odds-ratio = 2.21 ± 0.75, *p* = 0.021). Post hoc comparisons revealed that the probability to remember in the absence of feedback, significantly differed depending on whether the new-words were learned during the first or second half on the previous day (i.e., before or after participants started to receive feedback during learning; odds-ratio = 0.42, *p* = 0.013), while there were no order differences for new-words, which were learned while feedback was provided (*p* = 0.79; i.e., memory recognition was not affected if feedback was provided first or after the no-feedback condition). To assess whether this specific drop in recognition rates for congruent trials is also present when no feedback is provided througout the experiment, we re-analyzed our previous data^10^. Mirroring our analysis above, we estimated a mixed model of the likelihood to remember/forget the specific meaning (congruent trials) or that a new-word had an incongruent meaning. The fixed effects factors were congruence (congruent/incongruent), half (first half/second half) and their interaction, and we included a random intercept per subject. We did not observe a significant interaction of half and congruence as would be expected if this effect were specific for congruent trials (Bayes-Factor for the null-hypothesis: BF_01_ = 4.94), nor a significant main effect of half (BF_01_ = 7.22), suggesting that the above significant drop in recognition rates of congruent meanings in the feedback first group was not due to general effects like time or boredom.

In contrast, this pattern was absent for the new-words, which had been associated with two incongruent sentences (3-way interaction of feedback by order by congruence: *χ*^2^(1) = 4.74, *p* = 0.03). We observed a trend for feedback improving the memory for the incongruent new-words (for incongruent only, main effect of feedback: odds-ratio = 1.62 ± 0.45, *p* = 0.08), independently of when it was received (for incongruent only, interaction effect of feedback by order: odd-ratio = 0.85 ± 0.23, *p* = 0.55). The probability to correctly remember the incongruent new-words was also significantly above chance under all four conditions (all four *p’s* < 0.044).

In sum, this pattern of results suggests that, during our task, intrinsic- or extrinsic-generated DA-dependent signals interact and affect recognition memory. Importantly, participants benefited less from external feedback after being exposed to a period of self-regulated learning (internal DP-dependent signals) without external feedback.

### Experiment 2 (online)

To replicate our laboratory findings in another settings, we recruited seventy-three additional participants who completed a two-day online version of the task. The task structure was identical with the following exceptions: due to the online setting (a) participants now responded by typing their answers (instead of verbal responses in the lab experiment) during the meaning extraction session on day 1 and (b) they could proceed to the next trial only after typing an answer (i.e., we removed the option to answer “I don’t know”).

### Adding external feedback improves immediate word-meaning extraction

**Fig. 3:**
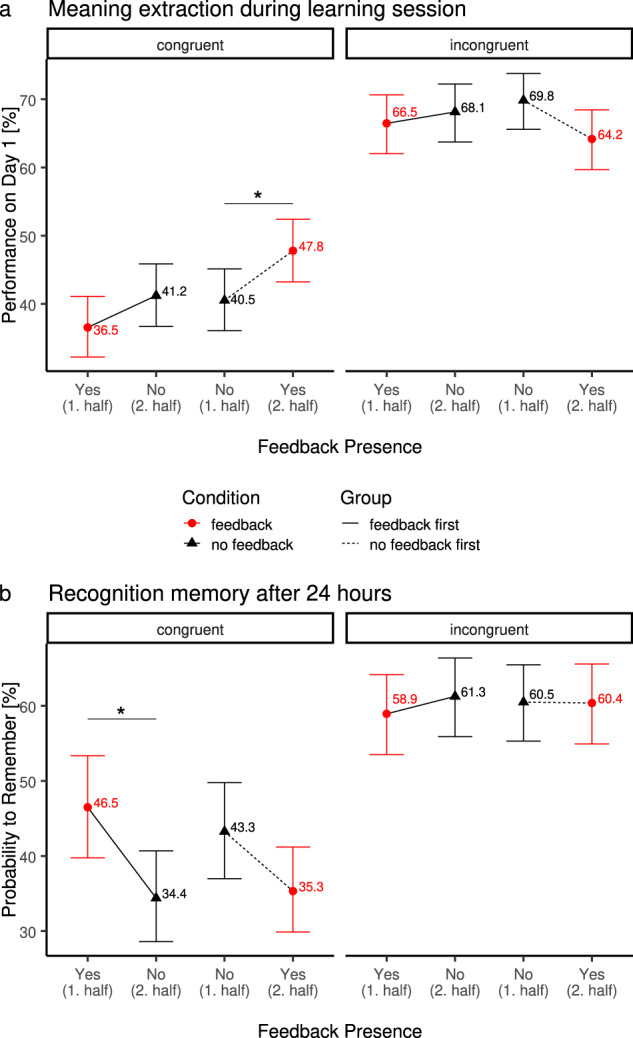
Behavioural results of Experiment 2 (online). Performance on new-word meaning extraction task on day 1 (**a**) and recognition memory on day 2 (**b**) in Experiment 2 (online). Trials with no congruent meaning (incongruent) are displayed on the right, trials with congruent meaning on the left, separately for feedback order (between subject effect; lines connect data points for the feedback first and no feedback first groups; full line: started with feedback; dotted line: started without feedback) and feedback presence (within-subject effect; red dot: feedback; black triangle: no feedback). Note that feedback presence is plotted in order of occurrence on *x*-axis (i.e., reversed feedback presence for the two groups). Lines between data points indicate identical within-group measures differing only in feedback presence. Error bars indicate 95% confidence interval. **a** Immediate performance during meaning extraction task (implicit learning). Across conditions incongruent trials (right side) showed a higher level of performance than congruent trials. Informative feedback improved performance for the group of participants who started off without feedback and started receiving feedback on their answers in the second half of the experiment, but only in the congruent condition (*p* = .02). **b** Recognition performance on day 2 as a function of congruence of meaning across sentences, feedback order and feedback presence (chance level = 33%). For incongruent trials, there were no significant different recognition rates across conditions. For congruent trials, removing feedback in the second half of the experiment lead to a substantial drop in recollection rates a day later (*p* = 0.03), whereas no effect was found if feedback was added in the second half of trials (i.e., if participants started the learning phase with no-feedback, *p* = 0.13).

Like in Experiment 1, we first assessed whether external feedback influenced immediate performance of meaning extraction (Fig. 3a) on day 1 using a generalized linear mixed model with the within-subject factors feedback (feedback/no feedback) and congruence (congruent/incongruent) and the between-subject factor order (feedback first/no feedback first), and a random intercept per subject.

On average, participants were able to correctly infer the meaning of new-words from the congruent contextual information (congruent trials: 41.5 ± 1.4% correct, see Fig. 2; this learning rate is similar, albeit somewhat smaller than in experiment 1. They were also able to correctly indicate when two sentences were incongruent (incongruent trials: 67.2 ± 1.3% correct; main effect of congruence: *χ*^2^(1) = 380.95, *p* < 0.0001, see Fig. 3a).

Importantly, receiving feedback in the second half lead to overall higher rates of extracting concrete word-meanings (feedback first 38.9% vs. no-feedback first 44.1%) but there was no difference in the rates of correctly identifying an absence of a single meaning across both sentences (interaction effect of congruence by order: *χ*^2^(1) = 4.25, *p* = 0.039). However, this effect was further qualified by feedback (three-way interaction effect of congruence by order by feedback: *χ*^2^(1) = 4.25, *p* = 0.039): Participants extracted concrete meanings best when they receiving feedback (47.8%) after having first done the task without any feedback (40.5%, odds-ratio = 1.34 ± 0.14, *p* = 0.005) while the group of participants who started with feedback did not change their performance significantly replicating the results from the first experiment. However, unlike in Experiment 1, we did not find any significant differences between incongruent trials.

### Removing external feedback decreases long-term memory for specific new-word meanings

To assess how this external feedback influenced memory consolidation processes, we next examined performance levels of the surprise memory recognition test 24 h later. In the online experiment, all participants performed a forced-choice recognition test (with 33% chance level, see Methods). Recognition rates were slightly smaller (see Fig. 3b), but comparable to those in experiment 1 and comparable in size with our previous work^10,11^ using the same task and memory test (congruent: 42.8%; incongruent: 36.5%).

To characterize how the presence of feedback affects recognition memory, we again focused our analysis on the subset of new-words, which were correctly answered on day 1, and could, thus, be either remembered or forgotten a day later. On average, participants more likely remembered the incongruent new-words (incongruent: 60.3% vs. congruent: 39.9%, main effect of congruence: *χ*^2^(1) = 119.71, *p* < 0.001) after 24 h.

Importantly, we found that while incongruent trials did not differ in their recognition rates as a function of feedback or order (all *p*’s > 0.53), the recognition rates for extracted word-meanings changed when comparing across both halves of the experiment as a function of whether and when feedback was provided (three-way interaction between congruence, order, and feedback: *χ*^2^(1) = 9.28, *p* = 0.002): Like in experiment 1, recognition rates dropped when feedback was removed (odds-ratio = 1.63 ± 0.30, *p* = 0.035) in the second half, but did not change when feedback was added later (odds-ratio = 0.69 ± 0.12, *p* = 0.13; congruent trials only: interaction effect of feedback by order: *χ*^2^(1) = 12.04, *p* < 0.001).

## Discussion

In this study, we probed the interplay of external (extrinsic) and internal (intrinsic) motivational signals on recognition memory by manipulating the presence of informative feedback in the context of learning of new word-meanings, in two independent experiments with different settings, laboratory and online, and two independent samples. We consistently found that these signals had distinct effects on immediate task performance during learning (day 1) and on more durable consolidation processes as evidenced by memory performance on day 2, and that those effects depended on what was being learned: a single, congruent word meaning of a new-word, or that a new-word had incongruent meanings across sentences. Importantly, we observed that the impact of an extrinsic signal (i.e., external feedback) on a memory of congruent word-meanings (congruent trials) depended on whether an intrinsically generated signal had been already established, that is, on whether self-regulated learning without feedback had had an opportunity to occur.

More specifically, during day 1, adding external feedback improved performance if it was provided in the second half, that is, after an initial, self-guided learning period without any feedback. This effect did not depend on what was to be learned—a specific meaning extracted from two congruent sentences or simply the fact that the sentences were incongruent in the laboratory experiment; in the online experiment, this effect was again observed for the congruent sentences pointing to the robustness of this effect, but not for the incongruent trials. Online experiments are by their nature less controlled, which could potentially account for this difference between the two experiments. However, a more specific explanation might be the potentially smaller impact of feedback in the online experiment, which lacked the social context: online, feedback was provided by an automatized program; in the laboratory, feedback was clearly provided by a human (the experimenter) in a non-automatized way. In accord, the source of feedback is a known moderator in the effectiveness of feedback, and social interactions compared to non-scial increase the effectiveness of feedback^32^ as does the actual partner of the interaction (student vs. teacher) with student-student feedback showing the largest effect sizes^34^, as used in the first laboratory experiment. From this perspective, adding weak feedback in the online experiment might not be enough to improve the performance of a process, which does not rely heavily on dopamine (i.e., incongruent trials), while a process, which recruits dopaminergic signaling even in a completely self-regulated setting^9–11^ (i.e., congruent trials), can be boosted even by weaker feedback.

Moreover, the performance of meaning extraction on day 1 improved substantially more when feedback was introduced in the second half of the task for congruent trials. Note that feedback itself could not directly enhance performance during the learning session, because it was given after each judgment about a specific new-word was made. Hence, the information about the past accuracy could not help to perform better on the subsequent trials when extracting other new meanings. One possibility is that adding performance feedback boosts motivation, leading to more attentional and cognitive resources being deployed. However, then performance should be lower on trials without feedback in general, and not in this time-dependent manner. Alternatively, performance might be boosted in tasks whose structure (also referred to as “learning set”^35^) had been already learned in a self-guided manner before the introduction of the extrinsic incentives. Then, the additional informative feedback about successful task performance could further strengthen these already established processing routines, leading to an increased performance on day 1.

In stark contrast to performance during the learning session on day 1, on the surprise memory-test performed by the participants a day later we observed markedly different pattern of effects for congruent trials. On day 1, participants more often correctly indicated that two sentences had been incongruent; on day 2, however, they were less likely to remember this fact about the new-words as compared to the likelihood of remembering a specific meaning of a new-word in the laboratory experiment. This large difference between congruent and incongruent new-words on memory recognition could be attributed to the well-known memory congruency effect^36–38^. Incorporating new information into long-term memory that is congruent with previous knowledge or “scheme” is easier than assimilating incongruent information. In this sense, when successfully discovering the meaning of a new-word, the semantic congruency of the previous two sentences might help to integrate this new trace into a coherent knowledge prior or scheme outside the SN/VTA-HP loop that might in turn reinforce encoding and later retrieval. This knowledge prior or scheme might be represented in prefrontal structures most likely inferior frontal gyrus (see ref. ^9^, Fig. 4) or superior frontal regions. However, we did not observe this large drop in recognition rates for incongruent new-words in the online experiment. This difference between online and laboratory settings might be potentially due to somewhat different strategies: When participants are unsure, they might default to guessing that it was an incongruent meaning and because of the less personal setting in the online experiment, they might be tempted to do so more often. However, it is important to note, that while the pattern of results in the incongruent trails changed, the pattern of results for the congruent trials remained unchanged across the two experiments pointing again at two at least partly independent processes. To address this more thoroughly, future research might assess the confidence during the learning phase on day 1. In the present work, we chose not to assess confidence during the learning phase, i.e., immediately after participants provided answers, because the duration between response and feedback can be an important factor in the efficacy of feedback^39^ and we aimed at providing feedback without any intervening psychological processes, and especially without explicitly focusing on meta-cognitive aspects like confidence.

On day 2 of the laboratory experiment, participants remembered slightly less that a new-word lacked a congruent meaning for those words that had been presented in trials with feedback, in comparison with those presented without feedback. Thus, when learning occurred in the absence of an intrinsically triggered DA-signal (incongruent trials), external rewards in form of informative feedback improved performance on the same day, as well as moderately improved memory recognition a day later, potentially by adding the DA-signal needed for LTP. Similar results have been obtained, for example, for visual working memory^40^ and learning of a list of words^41^. This can be seen in light of competence^6^, as feedback helps learners master the task at hand by facilitating evaluation and monitoring of learning performance^42–44^, thereby strengthening self-regulation^45,46^. Indeed, we observed at least a small enhancement for both memory and online performance in the presence of feedback in the laboratory settings. The lack of clear effects in the online experiment might be due to the less effective feedback as discussed above. In sum, our results are in line with the highly consistent main effect of extrinsic rewards^5^ on immediate performance but extend earlier observations by showing that the impact of feedback is dependent on the trial-specific DA-level.

In contrast to the incongruent trials, the results for congruent trials showed a more complex pattern. Specifically, the pattern of performance of meaning extraction (day 1) mimicked the one of the incongruent trials in the laboratory experiment, such that receiving feedback in the second half improved task success. While withdrawing feedback in the second half did not lead to a change in the performance on day 1, it profoundly affected recognition memory on day 2. Specifically, there was a drop in recognition rates of new-words that had to be learned without feedback after a preceding phase with feedback. It is at least conceivable that this differential effect of feedback between congruent and incongruent trials is due to the differential involvement of DA-signals for the congruent (potentially DA-dependent) and incongruent (potentially less DA-dependent) trials. In our previous work, we identified activity in the SN/VTA-HP loop^9,10^ in the congruent trials (but not incongruent trials) using functional neuroimaging and were able to alter the recognition rates using pharmacological manipulation using l-DOPA and risperidone, both thought to manipulate the dopaminergic system (though not exclusively). Note though that in other tasks, incongruent information can also activate (parts of) the SN/VTA-HP loop, for instance incongruent trials in a Stroop task^47^ and that different DA-signals are known to transmit distinct types of information^48^. A potentially important distinction to extensive work in the reinforcement learning tradition and our task is that learning the meaning of new words is typically associated with the declarative memory system^49^ while reinforcement learning tends to focus on procedural memory. The meaning-extraction task presented here is, from this perspective, more akin to single-shot learning or episodic memories and might require somewhat different computational approaches^50^ to clearly delineate the role of dopaminergic signaling.

Behaviorally, the present effect is akin to the well-known *undermining effect*^6^. Importantly, a drop in performance tied to the undermining effect has previously been related to a drop in activity in the VS and SN/VTA^51^. Since we observed this pattern only in DA-dependent (congruent) and not on DA-independent (incongruent) trials, our results suggest that a potential factor determining whether an undermining effect will be observed is the involvement of the SN/VTA-HP loop and the associated dopamine release^10,11^, revealing a possible connection between research on the undermining effect and computational approaches delineating different contributions of dopamine^52^.

A possible alternative explanation for this feedback-order dependence is the increasing levels of fatigue or boredom over the course of the experiment. Hence, with more trials more fatigue is present and adding feedback in the second half could counteract this fatigue-based effect. However, it is unclear whether fatigue or boredom would selectively affect congruent (DA-dependent) learning more than incongruent (DA-independent) learning. One could speculate that incongruent trials are more salient, as are errors and oddball stimuli^53^ more generally, and, thus, expect feedback to better counteract boredom or fatigue on those trials. If an increased salience of incongruent trials is indeed counteracting a general fatigue or boredom over the course of the experiment, this should also be the case in the complete absence of feedback. In a reanalysis of our previous data^10^, we did not observe any significant shifts in recognition rates over time, when no feedback was provided over the whole duration of the experiment, suggesting that fatigue or boredom are unlikely to account for the feedback-order dependence on congruent (DA-dependent) trials. Instead, learning the task structure^35^ in a self-regulated manner might be accompanied by a more robust, intrinsically generated DA release than learning the task with external feedback and the associated extrinsic DA release. From this perspective, adding external feedback in the second half leads to a slight decrease in memory, potentially due to the interaction of external and internal DA-signaling, while removal of the feedback leads to the removal of the main source of the DA signal (the feedback) while intrinsically guided DA-release is not established and, thus, prevents DA-dependent LTP from taking place. This would explain why the undermining effect is only observed in the DA-dependent (congruent) trials. Future neuroimaging work should be able to address this question.

Our results for the congruent trials are also in line with previous research proposing that insight (i.e., a sudden realization of something, in our paradigm, the meaning of the word to be learned) improves long-term memory^54–59^. Moreover, subsequent memory traces for answers to trivia questions are improved by providing monetary reward for correctly answered trivia questions and that this effect is driven by the uninteresting questions^60^. This suggests that the more interesting trivia questions trigger the SN/VTA-HP loop on their own^20^, similar to our congruent sentences and that the less interesting ones are DA-independent, akin to our incongruent trial, leading to the observed pattern. In line with our results, a stark difference was observed when comparing the effects of monetary reward between immediate and delayed memory tests (with the latter showing significant improvement)^60^. Moreover, a quantitative meta-analysis^5^ found that different measures of performance (quantitative vs. qualitative) show a differential link to intrinsic motivation and extrinsic rewards. They suggested that this might be due to those metrics being used for different types of tasks—quantitative ones for more boring and repetitive tasks, and qualitative metrics for tasks involving creativity and learning. Overall, this suggests that the effects of reward on immediate performance and memory consolidation processes can differ systematically. We propose that these distinct patterns might be caused by the involvement (or absence) of dopaminergic long-term potentiation processes, in line with the hippocampus-dependent memory consolidation model^12,61^.

These results have important implications for language learning in educational contexts as it provides a mechanistic explanation for the benefits of self-regulated learning and intrinsic motivation; concepts, which are implemented in Montessori schools among others^62,63^. They also have implications regarding clinical populations, as earlier studies with aphasic patients^64,65^ suggest that informative feedback enhances performance. In this vein, our results suggest that clinical patients (given that brain areas processing feedback are not too impaired^51,66–68^) engaging in DA-independent learning should profit more from paradigms in which feedback is provided. In contrast, our data do not support the notion that immediate task performance (unlike longer-term memory) will always deteriorate when feedback starts to be withheld^69^.

In sum, our work shows that intrinsic and extrinsic reward signals interact in a time-dependent manner and suggest that the precise pattern of the ensuing effects depends on whether they involve dopaminergic memory consolidation processes. Our results highlight the distinction between immediate performance during the learning session and consolidation-related memory retrieval processes and extend our understanding of how intrinsic motivation and extrinsic signals interact by taking the DA-dependent and DA-independent context into account. Together, this work suggests that future educational programs should incorporate self-regulated learning without feedback when intrinsic DA-dependent learning is bound to occur.

## Methods

### Experiment 1 (laboratory)

#### Participants

A group of sixty healthy, right-handed native German speakers took part in the experiment (*M*_age_ = 24.27 years, SD_age_ = 3.8, 40 females). Participants were recruited on campus of the Otto-von-Guericke University using emailing lists and poster announcements. Inclusion criteria were normal or corrected to normal vision, German as first language, and no self-reported history of mental illness. All volunteers gave their written informed consent approved by ethics commission of the Otto-von-Guericke University (approval 212/19) at the beginning of the experiment.

The original sample size was chosen to be ~30 participants per group (recall/recognition memory test, see below). This sample size was selected based on several criteria, including the recommendation that, in order to achieve 80% of power, at least 30 participants should be included in an experiment in which the expected effect size is medium to large^70^. In addition, we took into account the sample sizes of previous studies^9–11,71^ using this paradigm, which produced significant effects (range: between 24 and 40 participants). To account for potential drop-out due to the experiment spanning two consecutive days, we aimed to recruit 33 participants per group (based on previous experience in our lab of a 10% attrition rate). The final sample size was 31 participants in the free-recall group and 29 in the recognition test group (see below). Further mixed models were used for statistical analyses to maximize sensitivity^72^.

#### Materials

This study uses the same paradigm as our previous work^9–11^, except that we added performance-contingent feedback^6,73^ on half the trials. Stimuli were presented using Psychtoolbox (http://www.psychtoolbox.org) in MATLAB R2012b (The MathWorks, Inc., Natick, MA, USA).

Feedback consisted of either happy or sad smileys, indicating correct or incorrect responses, respectively (see Fig. 1). To control for the perception of faces^74,75^ and to match the trial duration, phase-transformed images of smileys^76,77^ were used in the no-feedback condition. Further, to prevent attributing a positive or negative meaning to any single phase-transformed stimulus, each scrambled feedback stimulus was displayed only once (Fig. 1; for more details on the materials & procedure see Supplementary Materials).

#### Procedure

As in our previous work, the experiment consisted of a learning phase (~2 h) and a memory-test phase (~30 min) a day later (*M* = 23 h, SD = 50 min).

#### Learning session

On the first day, participants read 80 pairs of sentences which ended in a new-word. Importantly, these pseudo-words were presented as part of either two congruent (40 pairs) or two incongruent sentences (40 pairs). In the congruent condition, a single meaning of a pseudo-word could be inferred from the two sentences (e.g., 1 “Every Sunday the grandmother went to the *jedin*” and 2. “The man was buried in the *jedin*”; here, “jedin” means graveyard and is congruent with both the first and second sentences). For incongruent sentence pairs no single coherent meaning could be inferred (e.g., 1. “Every night the astronomer watched the *heutil*” here moon is a possible meaning of “heutil; but 2. “In the morning break co-workers drink *heutil*”; here, coffee is now a possible meaning, which is inconsistent with the first sentence).

In order to mimic real-life contextual learning where novel words can be encountered at different times and in different contexts, first and second sentences ending in the same new-word, were presented separated in time and in sets of eight pairs (see Fig. 1a). In ten different blocks, participants were presented with four congruent and four incongruent pairs of sentences. In particular, participants first read all eight first sentences, each paired with a novel new-word; then, they read the eight second sentences paired with the same new-words, which were presented in randomized order relative to the first presentation. Immediately after finishing reading each second sentence and the associated new-word, participants were asked to verbally indicate the meaning of this new-word. This response was coded manually as correct/incorrect by the experimenter. Crucially, in half of the blocks, participants received informative feedback, and in the other half no-informative feedback (Fig. 1b). The order was counter-balanced across participants (feedback first/no feedback first). After the feedback screen, participants further rated their subjective state, along pleasantness and arousal using a 9-point Likert scale semantic differential. For pleasantness, participants rated how they pleasant they were feeling at this momemt, and for arousal how calm they were. On day 1 scales were given after the feedback to ensure that participants linked feedback to the meaning task and not to their subjective ratings, which could have happened if we had asked for self-evalutations prior to feedback.

#### Surprise memory test

Participants were told that the session on the second day would involve a personality evaluation, which would enable the investigation of the role of individual differences in personality on mood changes during reading. Instead, participants had to take a surprise memory test. Thirty-one participants underwent a cued free-recall memory test, while the remaining 29 participants took a recognition test.

In the free-recall task, participants were presented with all 80 new-words from the learning session and had to verbally indicate what its meaning was (for congruent new-words), that it had been paired with incongruent sentences (for incongruent new-words), or that they were unsure (“I don’t know”). Their responses were manually coded by the experimenter. Participants were uncertain for the majority of the new-words (53.8% of trials), and answered correctly only 18.2% of the trials, which were mostly the incongruent trials—only 4.4% of the congruent new-words were correctly recalled on the seconds day (i.e., between 1 and 2 new-words on average with only 12 participants showing any memory trace for any of the 40 congruent words at all). Owing to this low performance, floor effects are extremely likely and we did not analyze the free-recall data of day 2 any further.

In the recognition test, participants were presented with all 80 new-words from the learning session in random order and without their respective sentences or feedback, while given four response options: (1) the meaning evoked by the second sentence in which the new-word had been presented (i.e., the correct option for congruent trials, but a lure for incongruent trials), (2) with a lure (a randomly selected meaning from the remaining new-words presented during the learning phase), (3) an “incongruent” option (i.e., to signal that the new-word had no congruent meaning), and (4) an “I don’t know” response option to indicate that they were uncertain.

Additionally, participants were asked to provide pleasantness and arousal ratings after each word (using the same scales as on day 1). Further, participants rated their confidence that their answer had been correct (I am uncertain vs. certain) on a 9-Likert scale. All scales were poled in the same direction, with low ratings reflecting lower pleasantness, confidence and higher arousal.

### Experiment 2 (online)

#### Participants

A group of 73 healthy native German speakers completed the experiment (age and gender data were not collected based on German data protection law). Participants were recruited among the students of the Otto-von-Guericke University using a participant pool. Inclusion criteria were German as first language, and no self-reported history of mental illness. Participants were asked to use only desktop or laptop computers (no mobile) so that they could type their answers on a keyboard. All volunteers gave their written informed consent approved by ethics commission of the Otto-von-Guericke University (approval 212/19) at the beginning of the experiment.

Based our initial power calculation, a sample size of 30 participants should, in principle, be enough to replicate our results from experiment 1. However, we chose to increase (double) our target sample size as compared to experiment 1 since online contexts can have reduced effect sizes^78^. Based on typical dropout rates of roughly one third in online experiments^79^, we, thus, sought to recruit 90 participants. Nineteen participants started, but did not finish the experiment. Thus, the final sample included 36 participants in the “feedback first” group and 37 participants in the “no feedback first” group.

#### Materials

This experiment used the same materials as experiment 1. The task was programmed using the custom php script on the server and jspsych^80^ (version 7.0).

#### Procedure

As in experiment 1, the online experiment consisted of a learning session (~45 min) and a memory-test phase (~25 min) a day later (median = 24.8 h).

#### Learning session

The online learning session was almost identical to the learning session in experiment 1, except for the following changes: the blocks of 8 sentence pairs (see Fig. 1) were not balanced for congruence, but instead varied from 1/7 to 4/4 congruent/incongruent sentence pairs. This somewhat increases the ecological validity of the blocks, as real-life implicit learning opportunities will most likely not be perfectly balanced between congruent and incongruent new-word meanings and because some participants in Exp. 1 reported that they used this inferred information to shape their responses accordingly, although it was never provided by the experimenter. More importantly, participants gave written answers: for congruent word meanings, they wrote the meaning they thought the new-word had in both sentences; for incongruent meanings, they wrote “different” (German: “unterschiedlich”) indicating that they thought the new-word had incongruent meanings across the two sentences. All answers were converted to lower-case and then compared to the expected meanings, with any non-matching being counted as incorrect. Note that German capitalizes nouns, but in online contexts many German native speakers do not capitalize. Although strictly speaking grammatically incorrect, we chose to ignore this aspect, as it would not have made a difference if participants had responded verbally. Participant had to provide an answer of at least one character to proceed to read the next sentence, in contrast to experiment 1 in the laboratory, where they could remain silent to indicate “I don’t know” (see above) for 4 s to receive feedback.

#### Surprise memory test

The online memory test consisted of a forced-choice recognition test, like in experiment 1. Participants were shown the new-word on the top of the screen and pressed one of three buttons, two of which had specific meanings (i.e., the potential congruent meanings) or the word “different” (German: “unterschiedlich”). After pressing any of the three buttons, they proceeded to assess their confidence in their response as well as rate their subjective states on pleasantness and arousal. All three scales were implemented as sliders, with values ranging from 0 to 100. The statistical analysis and results are presented in the Supplementary Materials.

### Statistical analysis

All statistical analyses were conducted in R (version 3.6.1 & 4.2.1). To assess the performance during the learning session, we estimated a generalized linear mixed model with a logistic link function, for either correct or incorrect responses. This model is conceptually comparable to a logistic regression, but explicitly models the repeated measurement of responses from the same participant in multiple conditions^72^. Specifically, to account for the repeated-measures nature of the dataset we specified a random intercept per subject as a random factor. In addition, we included as fixed effects the main factors of interest, namely congruence (congruent/incongruent), feedback (feedback/no-feedback), and order (feedback first/no-feedback first), as well as all their interactions.

Analogously, for the recognition test we estimated a logistic mixed model for either remembering or forgetting the meaning of a new-word (i.e., correctly indicating a specific meaning for congruent trials and correctly answering “incongruent” for the incongruent trials), with the same fixed and random effects structure. To assess whether remembering performance was above chance, we conducted post hoc *t*-tests (two-sided) against chance level with a Bonferroni multiple-comparison correction.

For model estimation, we used the *mixed* function in the *afex* package^81^ (version 0.18), which in turn uses the *lme4* package^82^ (version 1.1) for the estimation of the models; *p*-values were computed using the Satterthwaite approximation of the degrees of freedom when assessing the significance of the fixed effects, as implemented by the *mixed* function. Importantly, mixed models account for different numbers of participants per factor level and still provide an unbiased estimate of effects^72^. Post hoc comparisons were calculated using the *emmeans* package^83^.

To assess whether the observed drop in recognition rates for the congruent word meanings learned without feedback in the second half (in the *feedback first* group) might be due to fatigue or boredom, we re-analyzed our previous data^10^. Like in the analysis above, we estimated a logistic mixed model of remembering vs. forgetting the specific word meaning (congruent trials) or correctly identifying a word as having had two incongruent meanings (incongruent trials) during the recognition test. Since no feedback was presented in our previous work, the model had congruence (congruent/incongruent), half(first half/second half), and their interaction as fixed effects, and a random intercept per subject. We estimated the model using the *brm* function in the *brms* package^84^ (version, 2.15.0) with the default, weakly informative priors (10,000 warm-up iterations, 10,000 sampling iterations, 4 chains) to be able to assess the Bayes-factor in favor of the null hypothesis using the function *bayes_factor* with the default settings.

See Supplementary Materials for the statistical analysis of the subjective ratings.

### Reporting summary

Further information on research design is available in the Nature Research Reporting Summary linked to this article.

## Supplementary information

Supplementary Material

Reporting Summary

## Data Availability

The behavioral data that support the findings of this study are available via OSF at 10.17605/OSF.IO/VJKP5.

## Code availability

The analysis code is available via OSF at 10.17605/OSF.IO/VJKP5.
