# Supplementary material for: Entering into a self-regulated learning mode prevents detrimental effects of feedback removal on memory
Source: NPJ Sci Learn. 2023 Jan 6;8:2. doi: 10.1038/s41539-022-00150-x (PMC9823107; doi:10.1038/s41539-022-00150-x)
Supplement: Supplementary file 1 — Supplementary Material [file 41539_2022_150_MOESM1_ESM.pdf]

Supplementary Materials for:
Entering into a self-regulated learning mode prevents detrimental effect of feedback removal on memory
by Vavra, Sokolovič, et al.,

Table of Contents

Notes on supplementary analyses....2
Experiment 1 (laboratory)....2
Pleasantness on day 1 ratings based on learning performance....4
Arousal on day 1 ratings based on day 1 performance....7
Pleasantness on day 1 based on remembered/forgotten....9
Arousal on day 1 based on remembered/forgotten....12
Pleasantness on day 2 based on remembered/forgotten....14
Arousal on day 2 based on remembered/forgotten....16
Confidence on day 2 based on remembered/forgotten....18
Experiment 2 (online)....20
Pleasantness on day 1 ratings based on learning performance....20
Arousal on day 1 ratings based on day 1 performance....22
Pleasantness on day 1 based on remembered/forgotten....25
Arousal on day 1 based on remembered/forgotten....27
Pleasantness on day 2 based on remembered/forgotten....30
Arousal on day 2 based on remembered/forgotten....32
Confidence on day 2 based on remembered/forgotten....34
Discussion....36

Notes on supplementary analyses

In addition to the learning (day 1) and memory (day 2) performance measures described in the main text, we also acquired subjective ratings. Since these measures are not central to our main claim, we present them in this supplementary materials for completeness and for comparison with previous studies using similar paradigms (Ripolles et al., 2014, 2016, 2018).

To facilitate the comparison with our previous work, we use the same nomenclature as in those publications. Specifically, congruent trials are now labeled as "M+" (a single meaning present), while incongruent trials are labeled as "M-" (a single meaning missing).

Further, to make the ANOVA tables shorter to read, we use the following labels for the conditions: "Order" now denotes feedback-order (feedback first/no feedback first), while congruence (congruent/incongruent) and feedback (feedback/no feedback) are the same as in the main text.

Going beyond the analysis in the main text which focused on performance, we can use the performance on both the meaning extraction task and the subsequent memory test on day 2 to further subdivide trials into correct/incorrect (based on the performance during the meaning extraction task on day 1 only) or remembered/forgotten/incorrect (factor called "RememForgot" below) which includes the information whether the new-word was correctly responded to during the memory test on day 2 (remembered=correct on both days; forgotten = correct on day 1, but incorrect on day 2; incorrect = already on day 1 incorrect).

All analyses were conducted, like in the main text, using R (version 4.2.1) by estimating mixed models with random intercepts for each subject. The fixed effects structure always included feedback (feedback/no feedback), congruence(congruent/incongruent), order(feedback first/no feedback first) and either correctness (correct/incorrect on day 1) or rememForgot(remembered/forgotten/incorrect), as well as their interactions. Models were estimated and factors assessed for significance using the *mixed* function from the *afex* package and significance tests were calculated using the likelihood ratio test.

Experiment 1 (laboratory)

For all analyses we sorted subjective ratings based on performance in the meaning extraction/memory task. Incorrect responses include erroneous and "I don't know" responses. On day 1, participants answered "I don't know" on 124 trials out of 4800 (2.58%). On day 2, participants answered "I don't know" in 1884 out of 4800 (39.25%) cases, when considering both free recall and recognition together; for the recognition test alone, they answered "I don't know" 549 out of 2320 trials (23.66%). Even though potentially being driven by slightly different processes, we pool the incorrectly answered trials with those where participants indicated "I don't

know" for these subsequent analyses. Note that we did not assess trial-based confidence on day 1; thus, a further grouping of "I don't know" responses was not possible.

To consider the ratings on day 1, we report two sets of analyses:

1. All 60 participants while considering their learning performance, but independent of their subsequent memory performance on the next day (*i.e.* dividing trials into correct/incorrect on day 1, regardless of whether they remember the word on day 2).

2. For only the participants who did the recognition test on day 2 (the recall test yielded too few correct trials on day 2), we split the ratings according to the combination of their performance on day 1 and day 2. Specifically, for correct words on day 1 we divided trials into those in which the specific meaning were correctly identified on day 2 (for new-words from congruent trials) or the lack of a congruent meaning was remembered on day 2 (for new-words of incongruent trials) and those in which this was forgotten.

Pleasantness on day 1 ratings based on learning performance

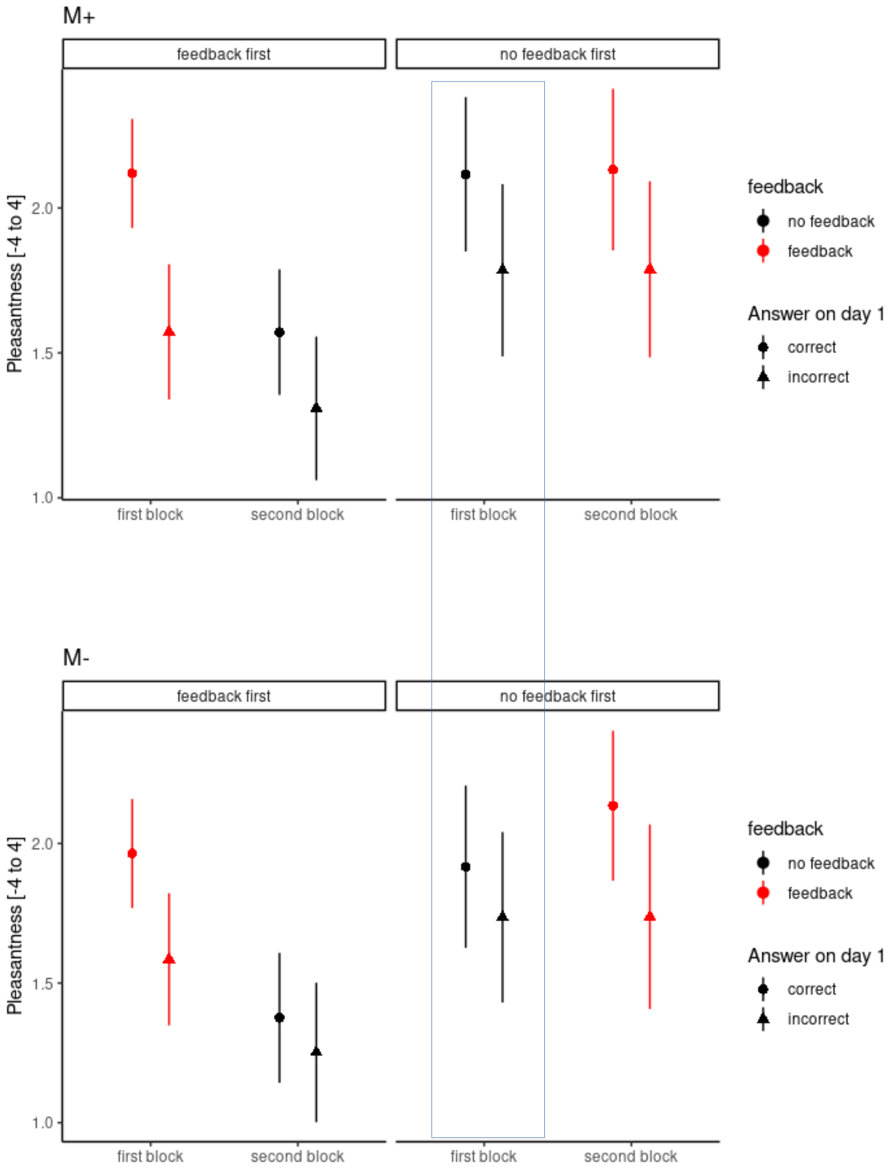

Supplementary Figure 1: Condition-specific group means and between-subject SEM for pleasantness ratings on day 1 sorted by performance on day 1 (correct/incorrect). Upper panel: M+ (dopamine-dependent trials). Lower panel: M- (dopamine independent trials). Left part of graphs: feedback first group; Right Part of graphs: no feedback first group. Framed area highlights the conditions similar to Ripolles et al., 2016.

Effect	df	Chisq	p
congruence	1	7.31 *	.014
feedback	1	64.53 **	.001
order	1	0.77	.402
correct	1	135.99 **	.001
congruence:feedback	1	1.63	.231
congruence:order	1	0.11	.737
feedback:order	1	47.59 **	.001
congruence:correct	1	4.55 *	.025
feedback:correct	1	12.70 **	.001
order:correct	1	1.20	.276
congruence:feedback:order	1	0.75	.390
congruence:feedback:correct	1	2.34	.118
congruence:order:correct	1	4.95 *	.030
feedback:order:correct	1	2.79	.103
congruence:feedback:order:correct	1	0.24	.632

Supplementary Table 1: Mixed model results for pleasantness ratings on day 1 based on performance on the same day. The mixed model was estimated with *congruence*, *feedback*, *feedback-order* and *correct/incorrect* as independent variables. A random intercept per subject was included. P-values were calculated with parametric bootstrapping. df = degrees of freedom; Chisq = chi square value; p = probability. “X:Y” denotes the interaction of X with Y.

Pleasantness ratings on day 1 revealed interactions of *Feedback* with *Order* and with *Correctness* in addition to the main effects of *Congruence*, *Feedback* and *Correctness*. Moreover, trial *Congruence* interacted with *Order* and *Correctness* (triple interaction). The interaction of *Feedback* with *Order* indicated that a drop in pleasantness ratings occurred if feedback was withdrawn whereas it slightly increased ratings if feedback was added later. These effects were independent of *Correctness* or *Order*. Additionally, differences in pleasantness ratings between correct and incorrect trials where larger for the congruent than incongruent trials. While this effect might be significant, it should be interpreted with caution since pleasantness ratings were obtained after performance feedback. Thus, the ratings after feedback may be more influenced by the explicit feedback than by the subjective evaluation of task performance as in our previous works (Ripolles et al., 2014,2016,2018). Likewise, the difference in pleasantness rating for correct and incorrect trials was slightly higher for congruent trials in the group starting with feedback, whereas the difference was slightly higher for M- trials in the group starting without feedback. Note again, that this comparison collapses across no-feedback and feedback trials, and thus might be difficult to interpret as it may contain influences from processes related to trial performance and feedback in some trials but not in others. Finally, we directly tested for a replication of the effect of pleasantness for the no-feedback trials (larger difference for correct-incorrect trials in the M+ but not the M- condition; Ripolles et al., 2014,2016,2018). The descriptive statistics indicate that pleasantness ratings were highest for the

M+ correct trials (see Supplementary Figure 1; framed region) despite the longer delay between response and pleasantness ratings in these experiments as compared to our previous studies (in the framed region, all pairwise comparisons with M+ correct trials: ps < .014). This effect was also observed for the no-feedback trials of the feedback first group.

Arousal on day 1 ratings based on day 1 performance

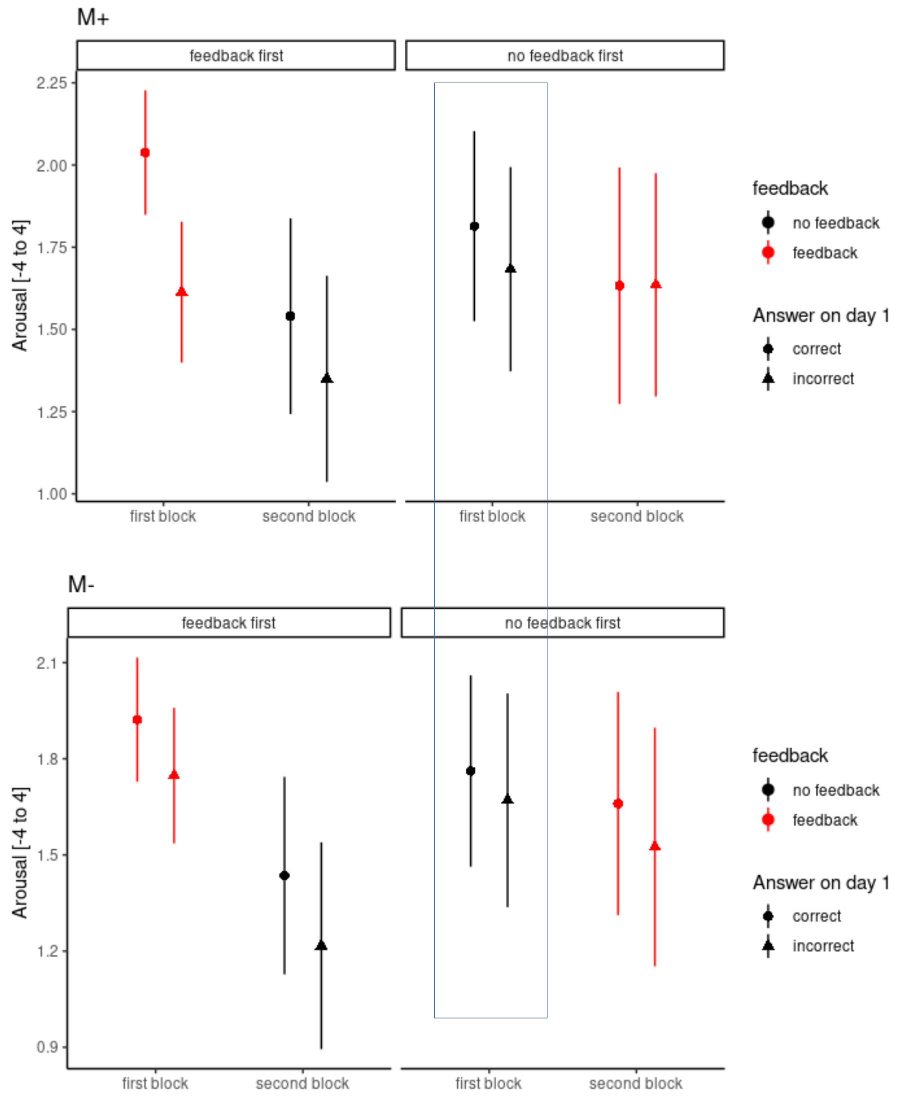

Supplementary Figure 2: Condition-specific group means and between-subject SEM for arousal ratings on day 1 sorted by performance on day 1 (correct/incorrect). Upper panel: M+ (dopamine-dependent trials). Lower panel: M- (dopamine independent trials). Left part of graphs: feedback first group; Right Part of graphs: no feedback first group. Framed area highlights the conditions similar to Ripolles et al., 2016.

Effect	df	Chisq	p
congruence	1	1.54	.221
feedback	1	20.83 **	.001
order	1	0.01	.915
correct	1	38.63 **	.001
congruence:feedback	1	1.27	.268
congruence:order	1	0.34	.554
feedback:order	1	67.57 **	.001
congruence:correct	1	1.58	.215
feedback:correct	1	0.03	.851
order:correct	1	1.45	.236
congruence:feedback:order	1	0.35	.561
congruence:feedback:correct	1	0.00	.962
congruence:order:correct	1	4.17 *	.042
feedback:order:correct	1	4.99 *	.022
congruence:feedback:order:correct	1	0.08	.779

Supplementary Table 2: Mixed model statistical results for arousal ratings on day 1 based on performance on the same day. Mixed models were estimated with *congruence*, *feedback*, *feedback-orde*r and *correct/incorrect* as independent variables. A random intercept per subject was included. P-values were calculated with parametric bootstrapping. df = degrees of freedom; Chisq = chi square value; p = probability. “X:Y” denotes the interaction of X with Y.

In addition to two main effects of Feedback and Correctness we observed an interaction of Feedback with order, and a triple interaction of Feedback with Order and Correctness plus again a triple interaction of Congruence with Order and correctness (see Supplementary Table 2 for details). The interaction of Feedback with Order was again caused by a drop in arousal ratings if feedback was withdrawn; further, arousal ratings to correct relative to incorrect trials were most pronounced in the group starting with feedback but only if feedback was provided yielding a significant triple interaction of Feedback with Order and Correctness. Moreover, the interaction of Congruence with Order and Correctness was driven by the difference in arousal ratings for correct vs. incorrect trials in congruent condition for the group starting with feedback. Finally, we directly tested for a replication of the null effect of arousal for the no-feedback trials (Ripolles et al., 2016,2018). The descriptive statistics indicate that arousal ratings were similar for the congruent/incongruent correct trials (see Supplementary Figure 2; framed region) resulting in no significant differences in arousal (main effect of congruence: $p > .22$, interaction of congruence and correctness: $p > .78$).

Pleasantness on day 1 based on remembered/forgotten

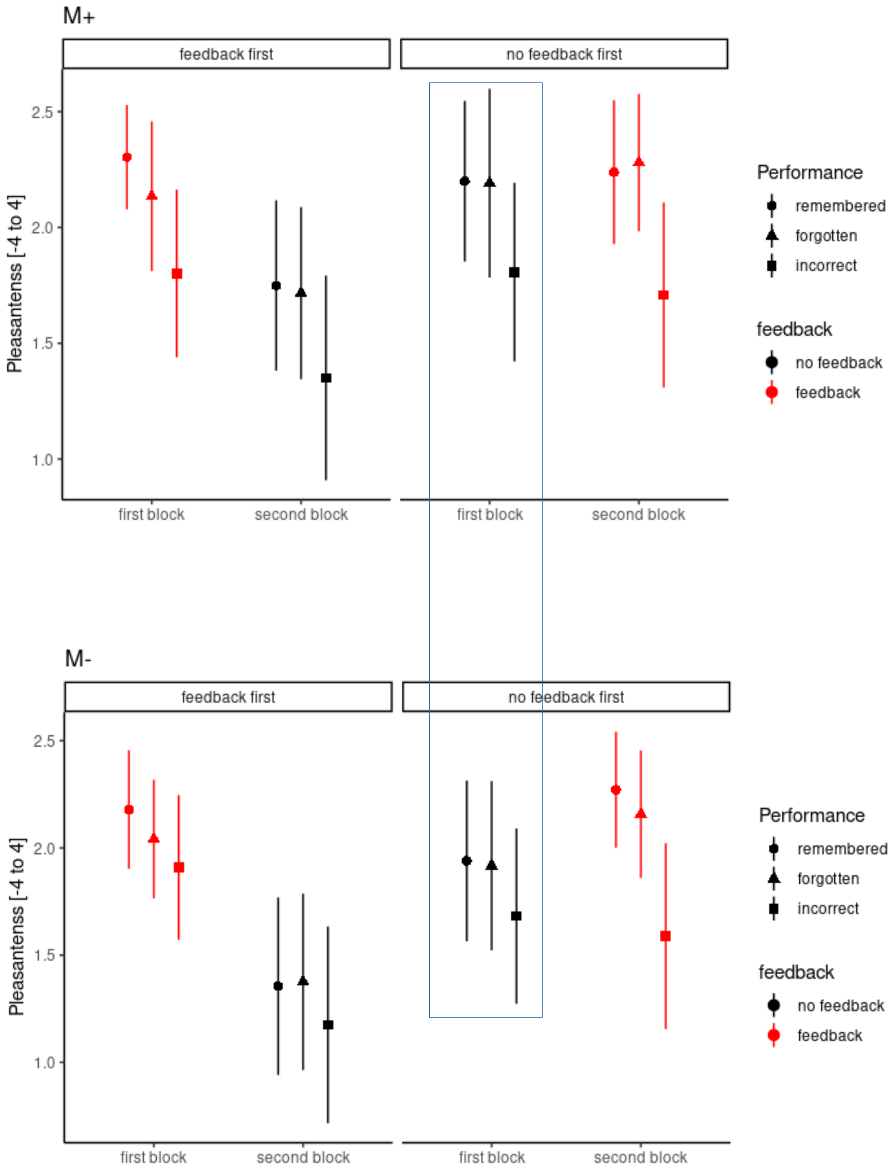

Supplementary Figure 3: Condition-specific group means and between-subject SEM for pleasantness ratings on day 1 sorted by performance on day 2 (remembered/forgotten/incorrect). Upper panel: M+ (dopamine-dependent trials). Lower panel: M- (dopamine independent trials). Left part of graphs: feedback first group; Right Part of graphs: no feedback first group. Framed area highlights the conditions similar to Ripollés et al., 2016.

Effect	df	Chisq	p
congruence	1	5.58 *	.018
feedback	1	62.16 **	.001
order	1	0.18	.680
rememForgot	2	56.18 **	.001
congruence:feedback	1	5.81 *	.013
congruence:order	1	0.10	.751
feedback:order	1	48.38 **	.001
congruence:rememForgot	2	3.48	.180
feedback:rememForgot	2	0.70	.736
order:rememForgot	2	5.31 +	.073
congruence:feedback:order	1	0.00	.951
congruence:feedback:rememForgot	2	3.20	.217
congruence:order:rememForgot	2	3.23	.217
feedback:order:rememForgot	2	1.43	.503
congruence:feedback:order:rememForgot	2	1.85	.402

Supplementary Table 3: Mixed ANOVA statistical results for pleasantness ratings on day 1 based on performance on day 2 (remembered/forgotten). Mixed models were estimated with *congruence*, *feedback*, *feedback-order* and *rememForgot* as independent variables. Note that Factor "*rememForgot*" has 3 levels: Remembered (correct on both days), forgotten (correct only on day 1) or incorrect (already on day1 incorrect). A random intercept per subject was included. P-values were calculated with parametric bootstrapping. df = degrees of freedom; Chisq = chi square value; p = probability. "X:Y" denotes the interaction of X with Y.

We further assessed the relationship of subjective ratings with memory performance by grouping the correct trials into those that were remembered on day 2 vs. those that were forgotten (note that this was only done for the group of subjects who performed the recognition task n=29, as free recall performance was too low). We observed that pleasantness ratings were different between remembered and incorrect trials in almost all conditions (significant main effect of *Remembered/Forgotten*; see Supplementary Table 3 and Supplementary Figure 3). Significant interactions were found for the factors *Feedback* with *Congruence*, *Feedback* with *Order* plus a trend for *Order* with *Remembered/Forgotten* (see Supplementary Table 3). The interaction of *Feedback* with *Order* was virtually identical to the one described above (drop in performance after feedback was withdrawn), whereas the interaction of *Feedback* with *Congruence* was due the ratings after feedback being equal for M+ trials and M- trials (both on average 2.05), while ratings in absence of feedback differed between M+ trials (on average 1.82) and M- trials (1.62, difference: p = .004). In addition, the trend towards *Order* and *Remembered/Forgotten* was caused by higher ratings for forgotten and remembered in the no feedback first group, as compared to the feedback first group. Finally, the pre-planned comparison of remembered vs. incorrect pleasantness ratings again indicated a larger difference for M+ than M- trials (p = .034).

Supplementary Materials for:

Entering into a self-regulated learning mode prevents detrimental effect of feedback removal on memory

by Vavra, Sokolovič, et al.,

Arousal on day 1 based on remembered/forgotten

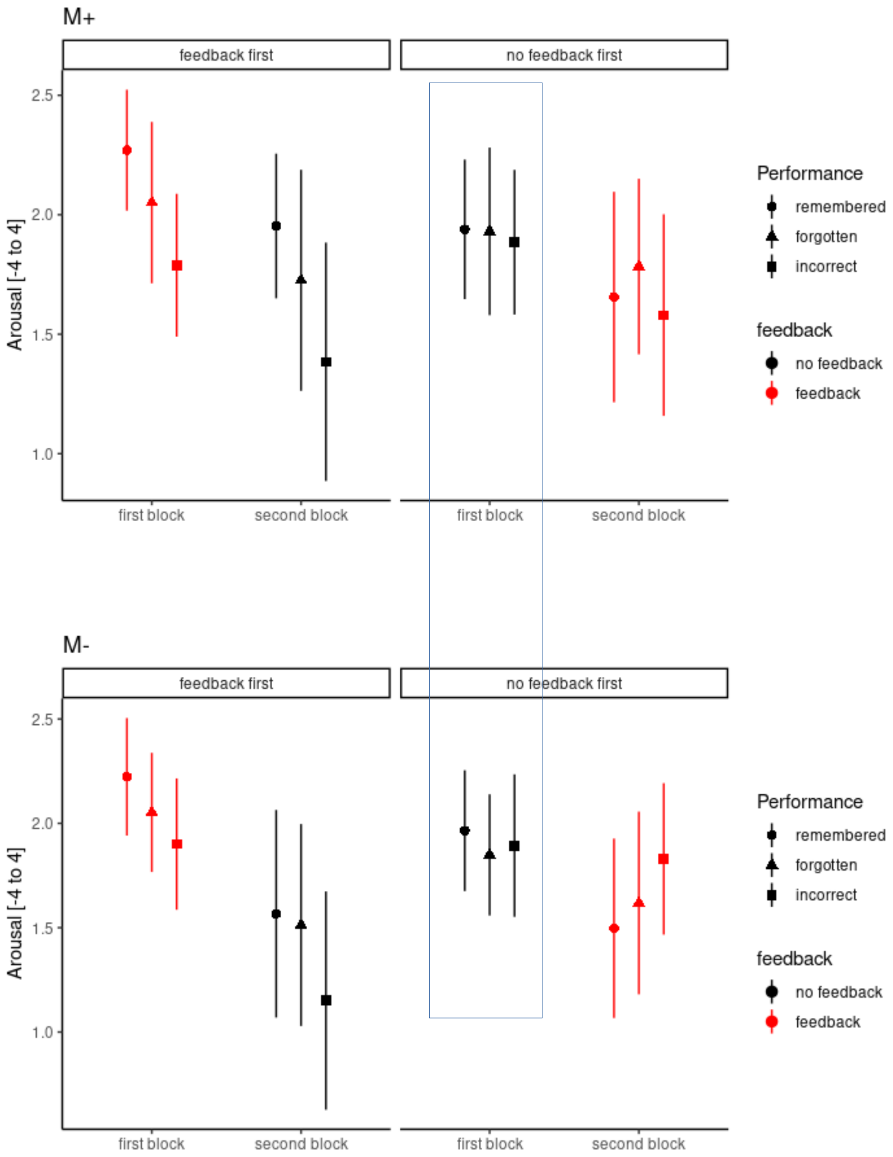

Supplementary Figure 4: Condition-specific group means and between-subject SEM for arousal ratings on day 1 sorted by performance on day 2 (remembered/forgotten/incorrect). Upper panel: M+ (dopamine-dependent trials). Lower panel: M- (dopamine independent trials). Left part of graphs: feedback first group; Right Part of graphs: no feedback first group. Framed area highlights the conditions similar to Ripolles et al., 2016.

Effect	df	Chisq	p
congruence	1	0.60	.424
feedback	1	7.27 **	.002
order	1	0.02	.899
rememForgot	2	9.08 *	.013
congruence:feedback	1	1.52	.212
congruence:order	1	0.18	.663
feedback:order	1	69.87 **	.001
congruence:rememForgot	2	2.74	.267
feedback:rememForgot	2	15.27 **	.001
order:rememForgot	2	5.04 +	.083
congruence:feedback:order	1	0.36	.564
congruence:feedback:rememForgot	2	2.47	.299
congruence:order:rememForgot	2	0.85	.656
feedback:order:rememForgot	2	0.85	.662
congruence:feedback:order:rememForgot	2	3.78	.164

Supplementary Table 4: Mixed ANOVA statistical results for arousal ratings on day 1 based on performance on day 2 (remembered/forgotten). Mixed models were estimated with *congruence*, *feedback*, *feedback-order* and *rememForgot* as independent variables. Note that Factor "*rememForgot*" has 3 levels: Remembered (correct on both days), forgotten (correct only on day 1) or incorrect (already on day1 incorrect). A random intercept per subject was included. P-values were calculated with parametric bootstrapping. df = degrees of freedom; Chisq = chi square value; p = probability. "X:Y" denotes the interaction of X with Y.

For arousal ratings on day 1 sorted by performance on day 2 we observed significant main effects of *Feedback* and *RememForgot* plus their interaction and the interactions of *Feedback* with *Order* and *rememForgot* with *Order*. The interaction of *Feedback* with *Order* was again due to the higher arousal ratings in the feedback first group when feedback was provided and lower arousal ratings without feedback relative to the no-feedback group. The interaction of *rememForgot with Order* was due to the monotonic increase in arousal ratings for incorrect to remembered trials in the feedback first group, which was not present in the no-feedback first group. Moreover, the interaction of *Feedback* with *RememForgot* was caused by the incorrect and forgotten trials having lower ratings in the absence of feedback (incorrect: difference 0.30, $p < .001$; forgotten: difference = 0.16, $p = .045$) as compared to those on trials with feedback, while the remembered trials did not differ significantly across feedback conditions ($p = .14$). Finally, the pre-planned comparison of remembered vs. incorrect arousal ratings again indicated no difference for M+ compared to M- trials (p>.63).

Pleasantness on day 2 based on remembered/forgotten

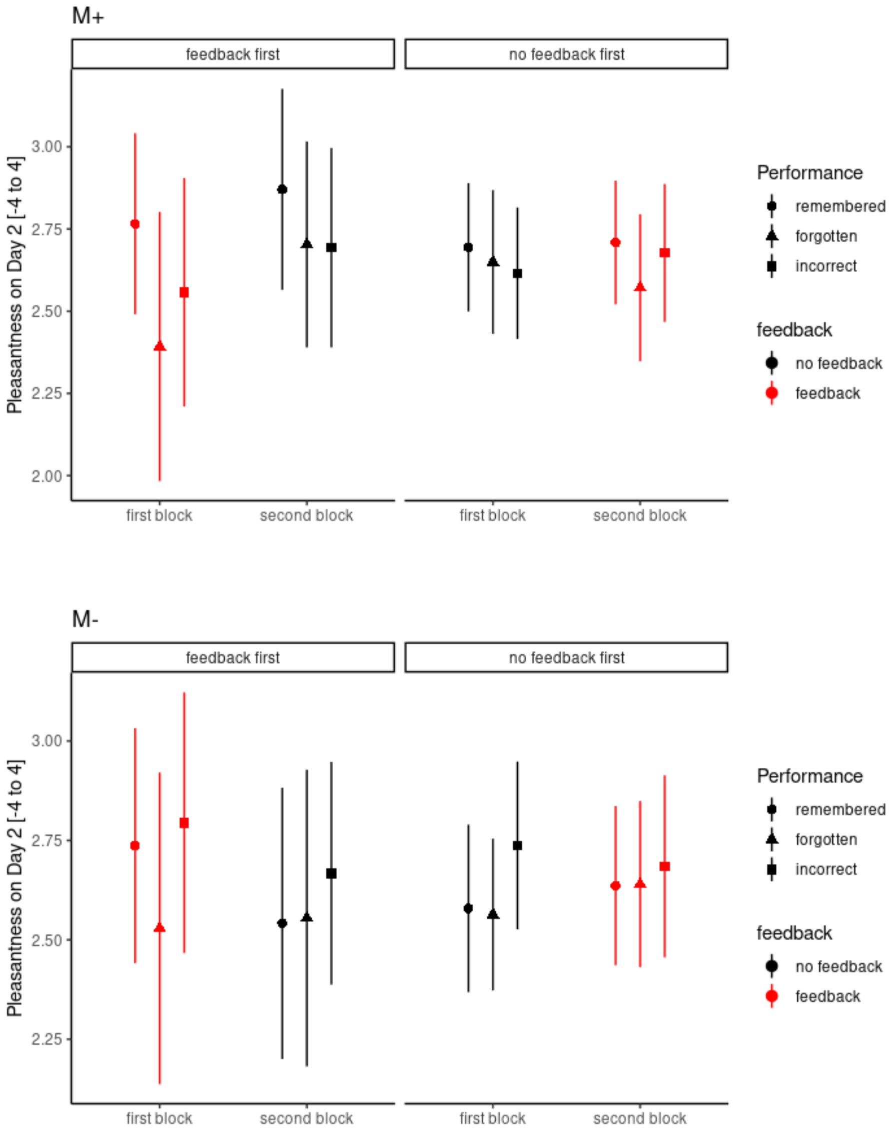

Supplementary Figure 5: Condition-specific group means and between-subject SEM for arousal ratings on day 2 sorted by performance on day 2 (remembered/forgotten/incorrect). Upper panel: M+ (dopamine-dependent trials). Lower panel: M- (dopamine independent trials). Left part of graphs: feedback first group; Right Part of graphs: no feedback first group.

**Effect**	**df**	**Chisq**	**p**
congruence	1	3.91 +	.053
feedback	1	0.00	.953
order	1	0.00	.978
rememForgot	2	5.70 *	.045
congruence:feedback	1	3.79 *	.046
congruence:order	1	1.76	.199
feedback:order	1	0.69	.405
congruence:rememForgot	2	16.09 **	.001
feedback:rememForgot	2	0.93	.638
order:rememForgot	2	1.08	.549
congruence:feedback:order	1	2.58	.113
congruence:feedback:rememForgot	2	0.44	.834
congruence:order:rememForgot	2	3.82	.158
feedback:order:rememForgot	2	1.51	.479
congruence:feedback:order:rememForgot	2	0.48	.788

Supplementary Table 5: Mixed ANOVA statistical results for pleasantness ratings collected on day 2 during the recognition test based on performance on day 2 (remembered/forgotten). Mixed models were estimated with *congruence*, *feedback*, *feedback-order* and *rememForgot* as independent variables. Note that Factor "*rememForgot*" has 3 levels: Remembered (correct on both days), forgotten (correct only on day 1) or incorrect (already on day1 incorrect). A random intercept per subject was included. P-values were calculated with parametric bootstrapping. df = degrees of freedom; Chisq = chi square value; p = probability. "X:Y" denotes the interaction of X with Y.

For pleasantness ratings on day 2, collected during the recognition test, we found a significant main effect of *rememForgot* plus a trend for *congruence,* their significant interaction (*congruence* x *rememForgot*), and the interaction of *congruence* with *feedback*. The interaction of congruence with feedback was due to higher pleasantness ratings for M+ trials without feedback but higher pleasantness ratings for M- trials with feedback on day 1 (note that no further feedback was provided on day 2). The interaction of *congruence* with *rememForgot* was reflected in highest pleasantness ratings for remembered M+ trials relative to forgotten and incorrect trails and lower ratings for remembered and incorrect M- trials relative to incorrect trials.

Arousal on day 2 based on remembered/forgotten

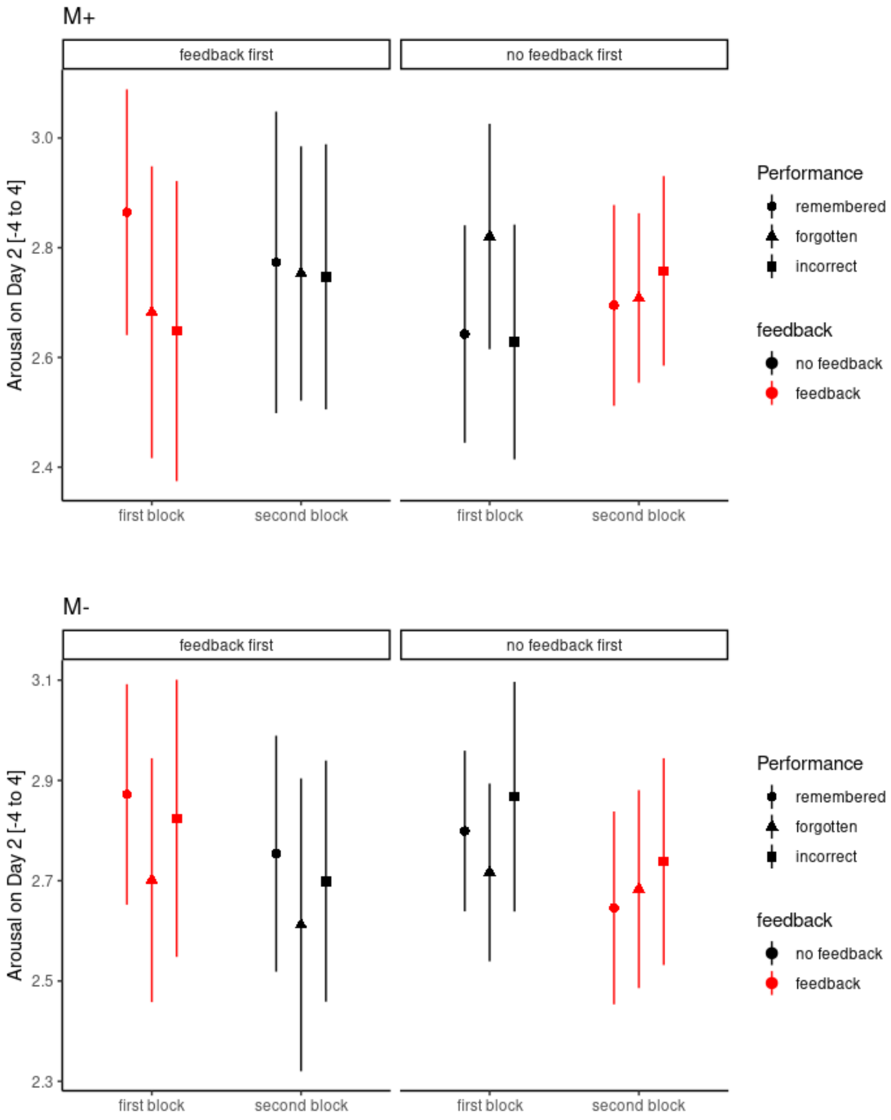

Supplementary Figure 6: Condition-specific group means and between-subject SEM for arousal ratings on day 2, collected during the recognition test, sorted by performance on day 2 (remembered/forgotten/incorrect). Upper panel: M+ (dopamine-dependent trials). Lower panel: M- (dopamine independent trials). Left part of graphs: feedback first group; Right Part of graphs : no feedback first group.

Effect	df	Chisq	p
congruence	1	0.86	.351
feedback	1	0.19	.686
order	1	0.03	.860
rememForgot	2	1.42	.510
congruence:feedback	1	0.02	.896
congruence:order	1	0.76	.391
feedback:order	1	2.43	.129
congruence:rememForgot	2	3.47	.185
feedback:rememForgot	2	1.05	.592
order:rememForgot	2	4.24	.133
congruence:feedback:order	1	2.05	.150
congruence:feedback:rememForgot	2	0.88	.666
congruence:order:rememForgot	2	2.12	.366
feedback:order:rememForgot	2	1.37	.518
congruence:feedback:order:rememForgot	2	3.67	.160

Supplementary Table 6: Mixed ANOVA statistical results for arousal ratings on day 2, collected during the recognition test, based on performance on day 2 (remembered/forgotten). Mixed models were estimated with *congruence*, *feedback*, *feedback-order* and *rememForgot* as independent variables. Note that Factor "*rememForgot*" has 3 levels: Remembered (correct on both days), forgotten (correct only on day 1) or incorrect (already on day1 incorrect). A random intercept per subject was included. P-values were calculated with parametric bootstrapping. df = degrees of freedom; Chisq = chi square value; p = probability. "X:Y" denotes the interaction of X with Y.

The arousal ratings on day 2, collected during the recognition test, did not cause any many effects or interactions (all p's >.13, see Supplementary Table 6).

Confidence on day 2 based on remembered/forgotten

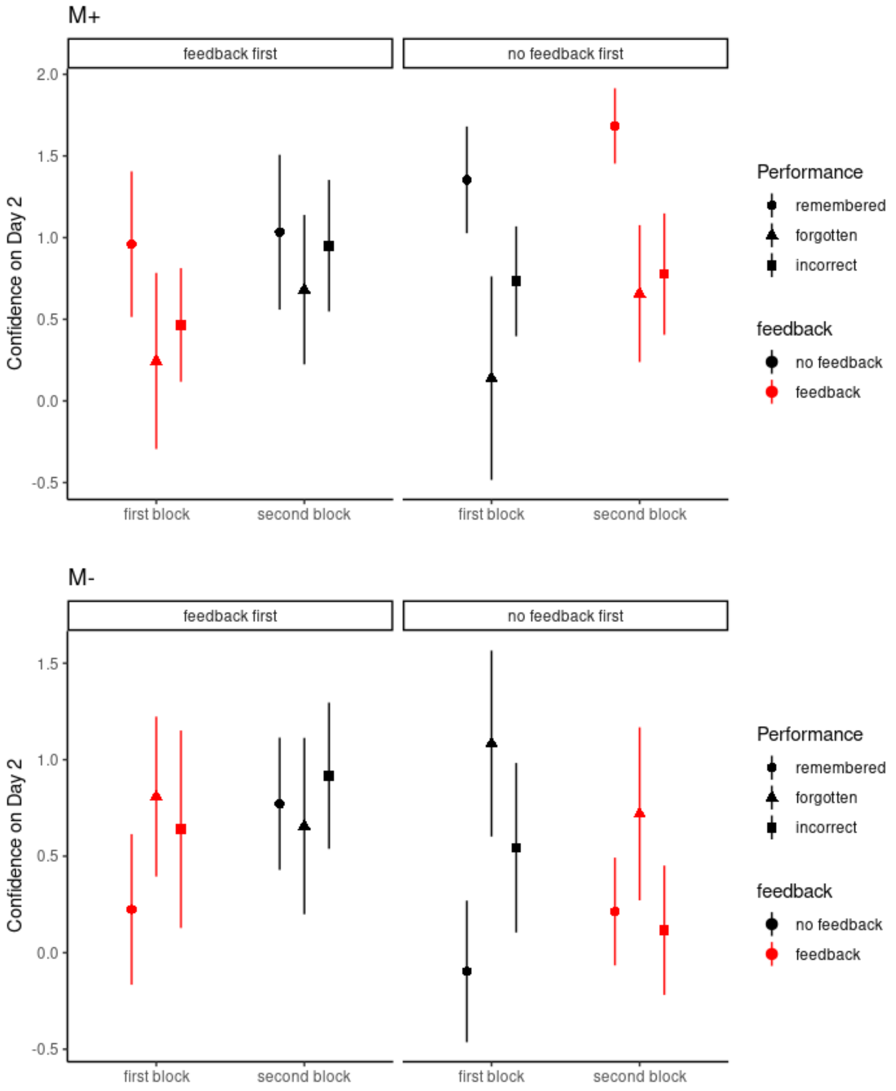

Supplementary Figure 7: Condition-specific group means and between-subject SEM for Confidence ratings on day 2, collected during the recognition test, sorted by performance on day 2 (remembered/forgotten/incorrect). Upper panel: M+ (dopamine-dependent trials). Lower panel: M- (dopamine independent trials). Left part of graphs: feedback first group; Right Part of graphs: no feedback first group.

Effect	df	Chisq	p
congruence	1	19.07 **	.001
feedback	1	0.86	.335
order	1	0.05	.844
rememForgot	2	6.25 *	.049
congruence:feedback	1	0.29	.591
congruence:order	1	2.16	.151
feedback:order	1	0.18	.666
congruence:rememForgot	2	46.64 **	.001
feedback:rememForgot	2	0.81	.659
order:rememForgot	2	0.24	.900
congruence:feedback:order	1	1.93	.187
congruence:feedback:rememForgot	2	0.92	.626
congruence:order:rememForgot	2	6.50 *	.046
feedback:order:rememForgot	2	1.55	.447
congruence:feedback:order:rememForgot	2	2.91	.186

Supplementary Table 7: Mixed ANOVA statistical results for confidence ratings on day 2, collected during the recognition test, based on performance on day 2 (remembered/forgotten). Mixed models were estimated with *congruence*, *feedback*, *feedback-order* and *rememForgot* as independent variables. Note that Factor "*rememForgot*" has 3 levels: Remembered (correct on both days), forgotten (correct only on day 1) or incorrect (already on day1 incorrect). A random intercept per subject was included. P-values were calculated with parametric bootstrapping. df = degrees of freedom; Chisq = chi square value; p = probability. "X:Y" denotes the interaction of X with Y.

For confidence ratings we found significant main effects of C*ongruence* and *RememForgot*, their interaction and a triple interaction of *congruence*, *rememForgot* with *order*. This interaction was due to highest confidence rating in the no feedback first group for remembered M+ trials (relative to forgotten and incorrect trials) and lowest confidence ratings for the forgotten trials; for M- trials this pattern was reversed. In the feedback first group the overall pattern was similar yet attenuated.

Experiment 2 (online)

Pleasantness on day 1 ratings based on learning performance

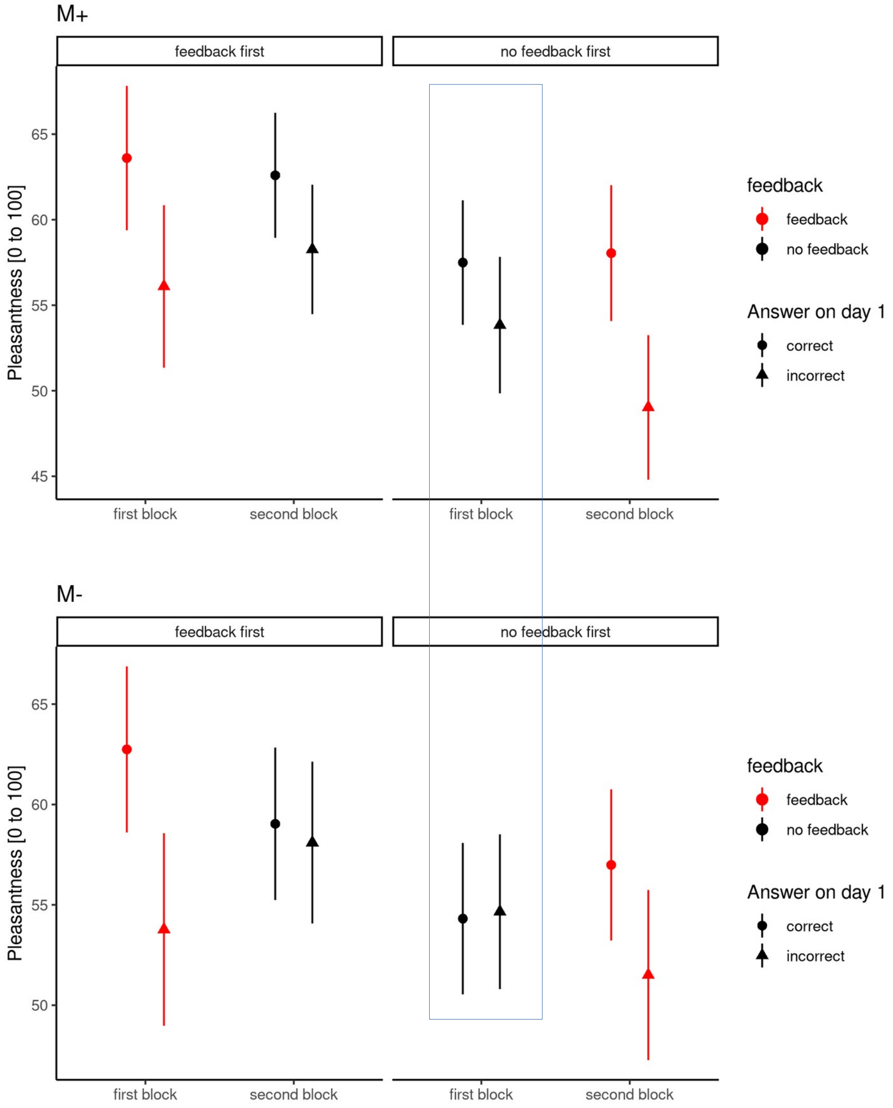

Supplementary Figure 8: Condition-specific group means and between-subject SEM for pleasantness ratings on day 1 sorted by performance on day 1 (correct/incorrect). Upper panel: M+ (dopamine-dependent trials). Lower panel: M- (dopamine independent trials). Left part of graphs: feedback first group; Right Part of graphs: no feedback first group. Framed area highlights the conditions similar to Ripolles et al., 2016.

Effect	df	Chisq	p
congruence	1	6.62 *	.014
feedback	1	2.69	.110
order	1	0.84	.375
correct	1	158.84 **	.001
congruence:feedback	1	0.35	.526
congruence:order	1	3.56 +	.059
feedback:order	1	3.36 +	.070
congruence:correct	1	6.26 *	.012
feedback:correct	1	43.29 **	.001
order:correct	1	0.83	.379
congruence:feedback:order	1	3.24 +	.061
congruence:feedback:correct	1	1.01	.317
congruence:order:correct	1	2.06	.148
feedback:order:correct	1	1.51	.212
congruence:feedback:order:correct	1	1.09	.314

Supplementary Table 8: Mixed model results for pleasantness ratings on day 1 based on performance on the same day. The mixed model was estimated with *congruence*, *feedback*, *feedback-order* and *correct/incorrect* as independent variables. A random intercept per subject was included. P-values were calculated with parametric bootstrapping. df = degrees of freedom; Chisq = chi square value; p = probability. “X:Y” denotes the interaction of X with Y.

For Pleasantness ratings on day 1, we found an interaction effect of *Congruence and Correctness*, in addition to main effects of *Correctness* and *Congruence*, such that for congruent trials the difference between correct and incorrect trials was larger as compared to incongruent trials. We also found an interaction of *Feedback by Correctness***,** such that correct trials were rated as more pleasant than incorrect trials in the feedback condition as compared to the no-feedback condition, suggesting that people incorporated the feedback into their pleasantness ratings similar to the first experiment.

Similarly to Ripollés et al., 2016, we observed higher pleasantness ratings for correct congruent trials as compared to incorrect congruent trials ($p = .0002$), but no significantly different for the incongruent trials ($p > .37$) in the no-feedback condition of the no-feedback first group (Framed area in Supplementary Figure 8).

Arousal on day 1 ratings based on day 1 performance

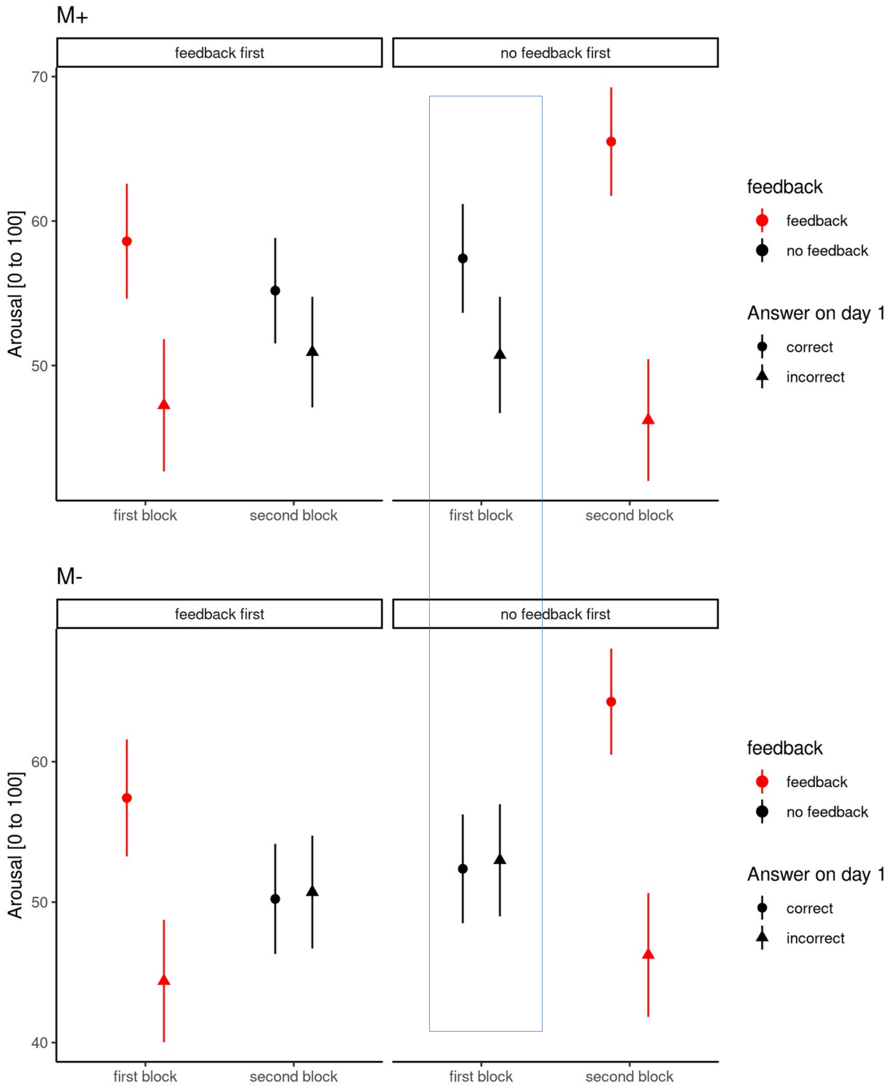

Supplementary Figure 9: Condition-specific group means and between-subject SEM for arousal ratings on day 1 sorted by performance on day 1 (correct/incorrect). Upper panel: M+ (dopamine-dependent trials). Lower panel: M- (dopamine independent trials). Left part of graphs: feedback first group; Right Part of graphs : no feedback first group. Framed area highlights the conditions similar to Ripolles et al., 2016.

Effect	df	Chisq	p
congruence	1	14.62 **	.001
feedback	1	3.31 +	.072
order	1	0.23	.651
correct	1	402.32 **	.001
congruence:feedback	1	0.03	.844
congruence:order	1	6.06 *	.012
feedback:order	1	2.62	.107
congruence:correct	1	9.54 **	.002
feedback:correct	1	174.68 **	.001
order:correct	1	12.46 **	.002
congruence:feedback:order	1	1.19	.249
congruence:feedback:correct	1	10.00 **	.003
congruence:order:correct	1	0.44	.510
feedback:order:correct	1	2.46	.108
congruence:feedback:order:correct	1	1.42	.243

Supplementary Table 9: Mixed model statistical results for arousal ratings on day 1 based on performance on the same day. Mixed models were estimated with *congruence*, *feedback*, *feedback-orde*r and *correct/incorrect* as independent variables. A random intercept per subject was included. P-values were calculated with parametric bootstrapping. df = degrees of freedom; Chisq = chi square value; p = probability. "X:Y" denotes the interaction of X with Y.

For the Arousal ratings on day 1, we found a significant interaction of *Congruence by Feedback by Correctness* (three-way interaction), such that the difference in arousal for correct and incorrect responses was smallest for the incongruent trials without feedback, largest for congruent and incongruent trials with feedback, with congruent trials without feedback falling in between those two. This three-way interaction qualified the simpler two-way interactions of *Correctness with Feedback* and *Congruence*, as well as the main effects of *Congruence* and *Correctness.* We also observed interactions of *Order* with *Correctness* and C*ongruence.* The interaction with *Correctness* was due to the difference in arousal between correct and incorrect trials being larger in the group of participants who started without feedback first, as compared to the group who started with feedback. The interaction with *Congruence* indicated that the group who started with feedback rated the incongruent trials as less arousing than the congruent trials, or any trials by the group who started without feedback. Note that in both experiments feedback and correctness interacted; however, in experiment 1 this pattern was further split by order whereas in experiment 2 it was further split by congruence. Unlike Ripollés et al., 2016, we observed higher arousal ratings for correct congruent trials as compared to incorrect congruent trials ($p < .0001$), but not significantly different for the incongruent trials ($p > .21$) in the no-feedback condition of the no-feedback first

group (Framed area in Supplementary Figure 9). Hence, the overall pattern was to some extent different from experiment 1, which might be due the online vs. laboratory settings.

Pleasantness on day 1 based on remembered/forgotten

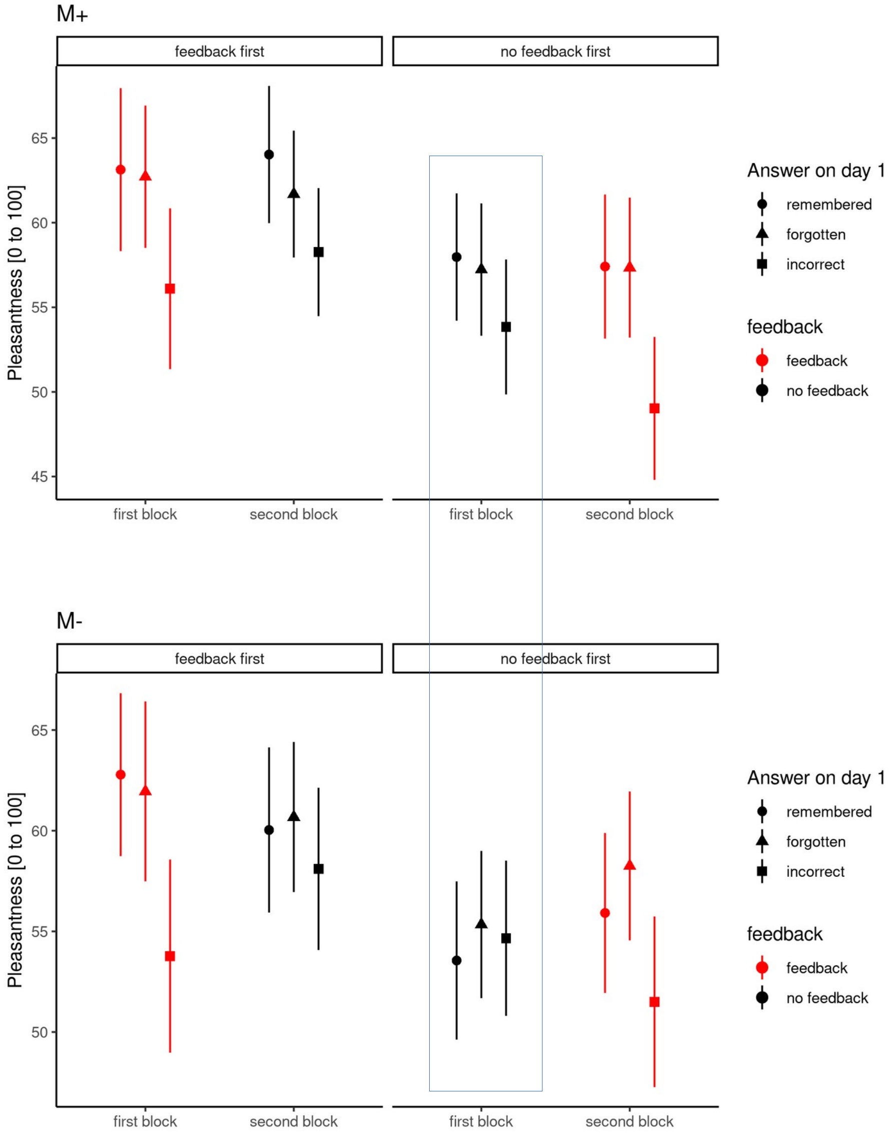

Supplementary Figure 10: Condition-specific group means and between-subject SEM for pleasantness ratings on day 1 sorted by performance on day 2 (remembered/forgotten/incorrect). Upper panel: M+ (dopamine-dependent trials). Lower panel: M- (dopamine independent trials). Left part of graphs: feedback first group; Right Part of graphs : no feedback first group. Framed area highlights the conditions similar to Ripolles et al., 2016.

Effect	df	Chisq	p
congruence	1	11.13 **	.001
feedback	1	0.17	.683
order	1	0.9	.360
RememForgot	2	164.72 **	.001
congruence:feedback	1	1.11	.269
congruence:order	1	1.85	.190
feedback:order	1	4.09 +	.051
congruence:RememForgot	2	9.54 **	.009
feedback:RememForgot	2	43.14 **	.001
order:RememForgot	2	1.29	.525
congruence:feedback:order	1	1.09	.266
congruence:feedback:RememForgot	2	2	.365
congruence:order:RememForgot	2	1.97	.353
feedback:order:RememForgot	2	4.65 +	.092
congruence:feedback:order:RememForgot	2	1.91	.385

Supplementary Table 10: Mixed ANOVA statistical results for pleasantness ratings on day 1 based on performance on day 2 (remembered/forgotten). Mixed models were estimated with *congruence*, *feedback*, *feedback-order* and *rememForgot* as independent variables. Note that Factor "*rememForgot*" has 3 levels: Remembered (correct on both days), forgotten (correct only on day 1) or incorrect (already on day1 incorrect). A random intercept per subject was included. P-values were calculated with parametric bootstrapping. df = degrees of freedom; Chisq = chi square value; p = probability. "X:Y" denotes the interaction of X with Y.

We further assessed the relationship of subjective ratings with memory performance by grouping the correct trials into those that were remembered on day 2 vs. those that were forgotten. For pleasantness ratings, we found significant interactions of *RememForgot* with *Feedback* such that incorrect trials were rates as more pleasant in trials without feedback, as compared to incorrect trials with feedback. In addition, the interaction between *RememForgot* and *Congruence* indicated that the difference in ratings for correct and incorrect trials was larger in congruent trials than in incongruent trials.

Unlike Ripollés et al. (2016), we did not observe a significant difference between remembered and forgotten trials in the first half of the experiment in the no-feedback first group but qualitatively replicated the findings of Experiment 1.

Arousal on day 1 based on remembered/forgotten

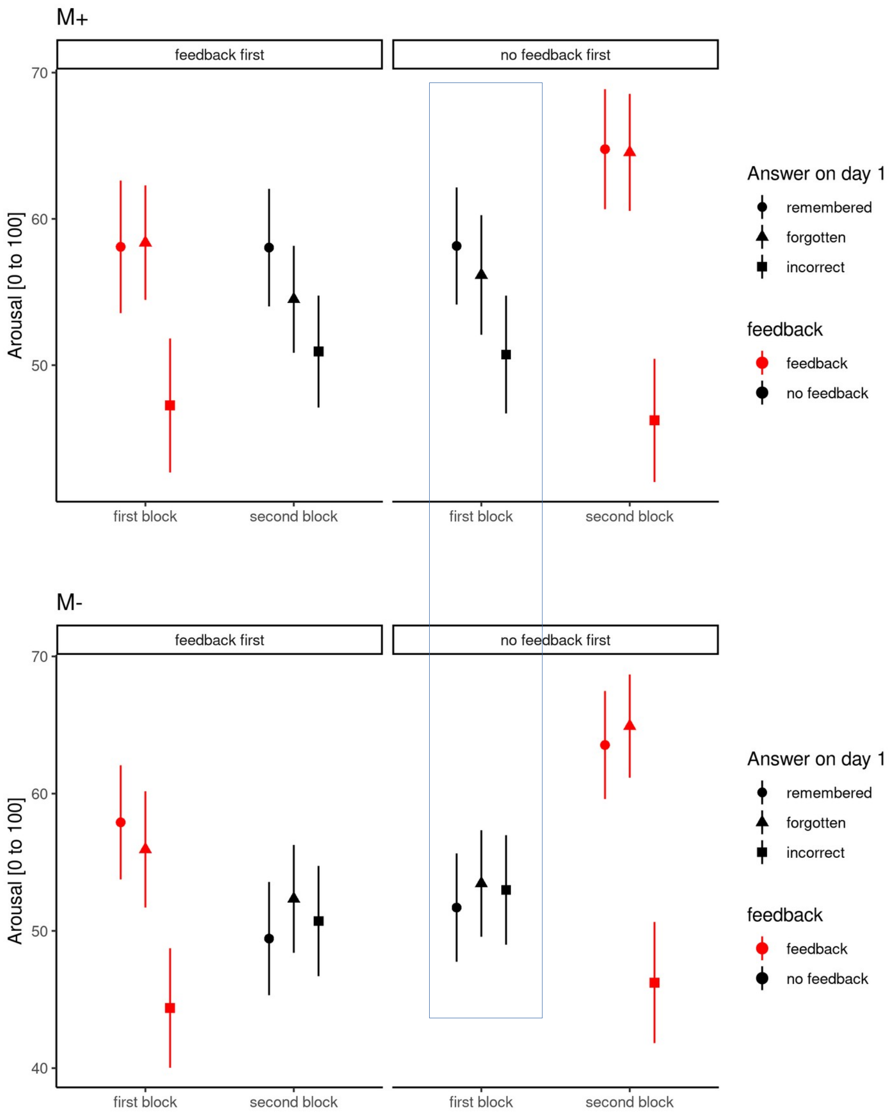

Supplementary Figure 11: Condition-specific group means and between-subject SEM for arousal ratings on day 1 sorted by performance on day 2 (remembered/forgotten/incorrect). Upper panel: M+ (dopamine-dependent trials). Lower panel: M- (dopamine independent trials). Left part of graphs: feedback first group; Right Part of graphs : no feedback first group. Framed area highlights the conditions similar to Ripolles et al., 2016.

**Effect**	**df**	**Chisq**	**p**
congruence	1	22.79 **	.001
feedback	1	36.03 **	.001
order	1	0.35	.571
RememForgot	2	411.72 **	.001
congruence:feedback	1	1.56	.193
congruence:order	1	4.31 *	.032
feedback:order	1	4.76 *	.040
congruence:RememForgot	2	16.59 **	.001
feedback:RememForgot	2	172.12 **	.001
order:RememForgot	2	12.54 **	.003
congruence:feedback:order	1	0.06	.782
congruence:feedback:RememForgot	2	10.22 **	.009
congruence:order:RememForgot	2	0.49	.778
feedback:order:RememForgot	2	6.07 *	.049
congruence:feedback:order:RememForgot	2	2.29	.326

Supplementary Table 11: Mixed ANOVA statistical results for arousal ratings on day 1 based on performance on day 2 (remembered/forgotten). Mixed models were estimated with *congruence*, *feedback*, *feedback-order* and *rememForgot* as independent variables. Note that Factor "*rememForgot*" has 3 levels: Remembered (correct on both days), forgotten (correct only on day 1) or incorrect (already on day1 incorrect). A random intercept per subject was included. P-values were calculated with parametric bootstrapping. df = degrees of freedom; Chisq = chi square value; p = probability. "X:Y" denotes the interaction of X with Y.

For the arousal ratings, we found a significant three-way interaction between *Feedback*, *RememForgot,* and *Order*. Pairwise post-hoc tests revealed that this interaction was driven by the no-feedback condition differing between the two *Order*-groups: In the group who started with feedback, the remembered, forgotten or incorrect did not significantly differ pairwise while receiving no feedback in the second half of the experiment; in contrast, the group who started without feedback, the ratings for incorrect answers were significantly lower than on remembered and forgotten trials in the absence of feedback. However, these differences should be interpreted with caution since they collapsed across *Congruence*, and we also observed a three-way interaction between *Feedback, RememForgt* and *Congruence*. Post-hoc test revealed that this interaction was driven by ratings in the no-feedback blocks, where we observed higher ratings for remembered, forgotten as compared to incorrect trials in the congruent, but not significantly different for incongruent trials. In the feedback block, this difference for remembered, forgotten and incorrect

trials was present for both congruent and incongruent trials (interaction effect for *Feedback* by *RememForgot*).

Pleasantness on day 2 based on remembered/forgotten

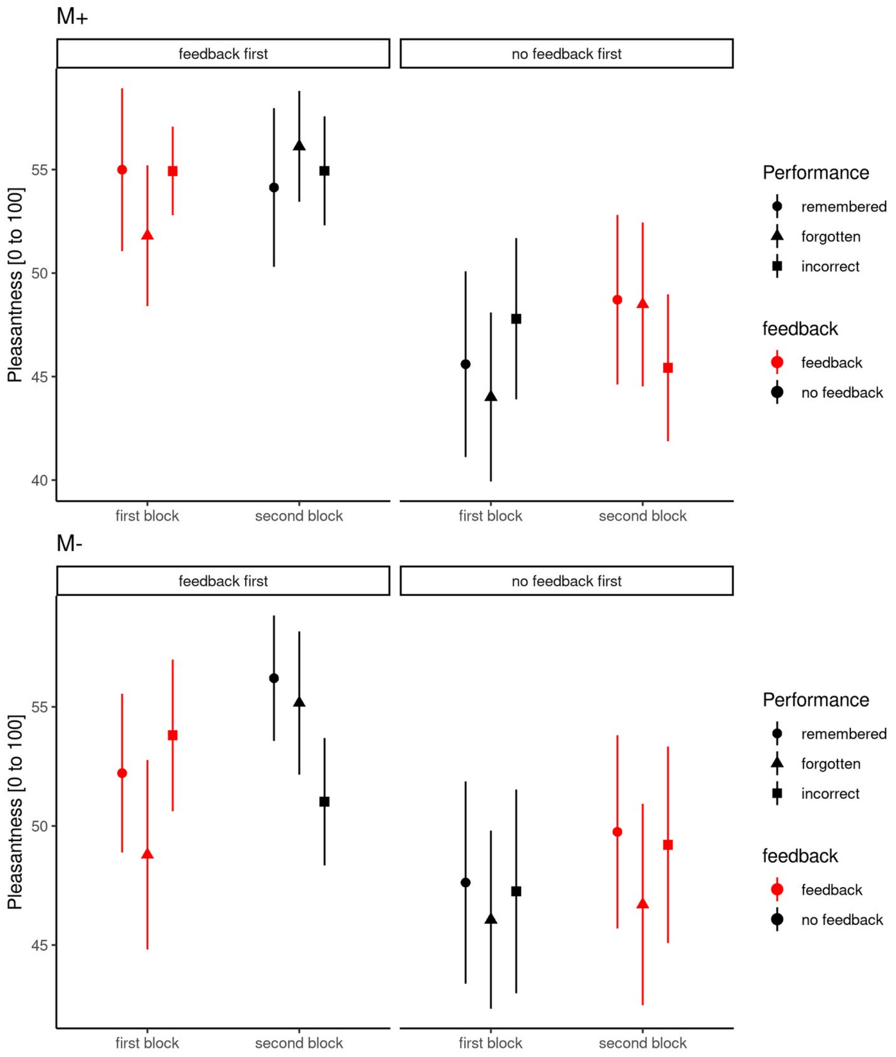

Supplementary Figure 12: Condition-specific group means and between-subject SEM for pleasantness ratings on day 2, collected during the recognition test, sorted by performance on day 2 (remembered/forgotten/incorrect). Upper panel: M+ (dopamine-dependent trials). Lower panel: M- (dopamine independent trials). Left part of graphs: feedback first group; Right Part of graphs: no feedback first group.

Effect	Df	Chisq	P
Congruence	1	0.95	.336
feedback	1	0.67	.426
Order	1	2.42	.120
RememForgot	2	4.47	.119
congruence:feedback	1	0.05	.800
congruence:order	1	1.91	.181
feedback:order	1	0.86	.362
congruence:RememForgot	2	0.77	.660
feedback:RememForgot	2	1.66	.454
order:RememForgot	2	1.56	.451
congruence:feedback:order	1	0.01	.919
congruence:feedback:RememForgot	2	3.54	.179
congruence:order:RememForgot	2	2.33	.283
feedback:order:RememForgot	2	2.03	.348
congruence:feedback:order:RememForgot	2	2.29	.235

Supplementary Table 12: Mixed ANOVA statistical results for pleasantness ratings on day 2 collected during the recognition test, based on performance on day 2 (remembered/forgotten). Mixed models were estimated with *congruence*, *feedback*, *feedback-order* and *rememForgot* as independent variables. Note that Factor "*rememForgot*" has 3 levels: Remembered (correct on both days), forgotten (correct only on day 1) or incorrect (already on day1 incorrect). A random intercept per subject was included. P-values were calculated with parametric bootstrapping. df = degrees of freedom; Chisq = chi square value; p = probability. "X:Y" denotes the interaction of X with Y.

All analyses of day 2 included the incorrect trials (unlike the remembered forgotten analysis on day 1) as they had not been analyzed previously. The pleasantness ratings on day 2 did not cause any many effects or interactions (all p's >.119, see Supplementary Table 12).

Arousal on day 2 based on remembered/forgotten

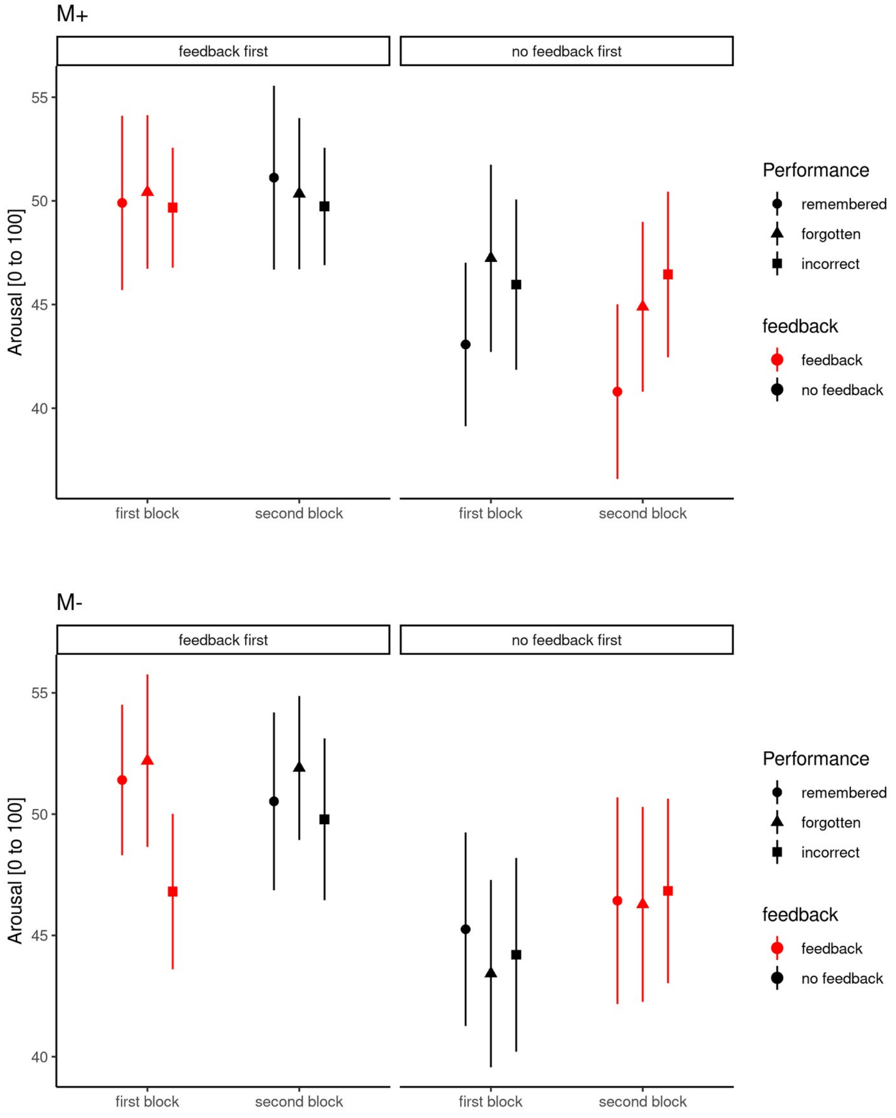

Supplementary Figure 13: Condition-specific group means and between-subject SEM for arousal ratings on day 2 collected during the recognition test, sorted by performance on day 2 (remembered/forgotten/incorrect). Upper panel: M+ (dopamine-dependent trials). Lower panel: M- (dopamine independent trials). Left part of graphs: feedback first group; Right Part of graphs: no feedback first group.

Effect	df	Chisq	p
congruence	1	0.00	.966
feedback	1	0.01	.920
order	1	1.25	.281
RememForgot	2	0.51	.817
congruence:feedback	1	1.97	.151
congruence:order	1	0.04	.839
feedback:order	1	0.47	.509
congruence:RememForgot	2	0.60	.734
feedback:RememForgot	2	0.47	.809
order:RememForgot	2	0.83	.668
congruence:feedback:order	1	1.19	.244
congruence:feedback:RememForgot	2	0.96	.630
congruence:order:RememForgot	2	3.26	.170
feedback:order:RememForgot	2	0.30	.852
congruence:feedback:order:RememForgot	2	0.43	.791

Supplementary Table 13: Mixed ANOVA statistical results for arousal ratings on day 2 collected during the recognition test, based on performance on day 2 (remembered/forgotten). Mixed models were estimated with *congruence*, *feedback*, *feedback-order* and *rememForgot* as independent variables. Note that Factor "*rememForgot*" has 3 levels: Remembered (correct on both days), forgotten (correct only on day 1) or incorrect (already on day1 incorrect). A random intercept per subject was included. P-values were calculated with parametric bootstrapping. df = degrees of freedom; Chisq = chi square value; p = probability. "X:Y" denotes the interaction of X with Y.

The arousal ratings on day 2 did not cause any many effects or interactions (all p's >.17, see Supplementary Table 13).

Confidence on day 2 based on remembered/forgotten

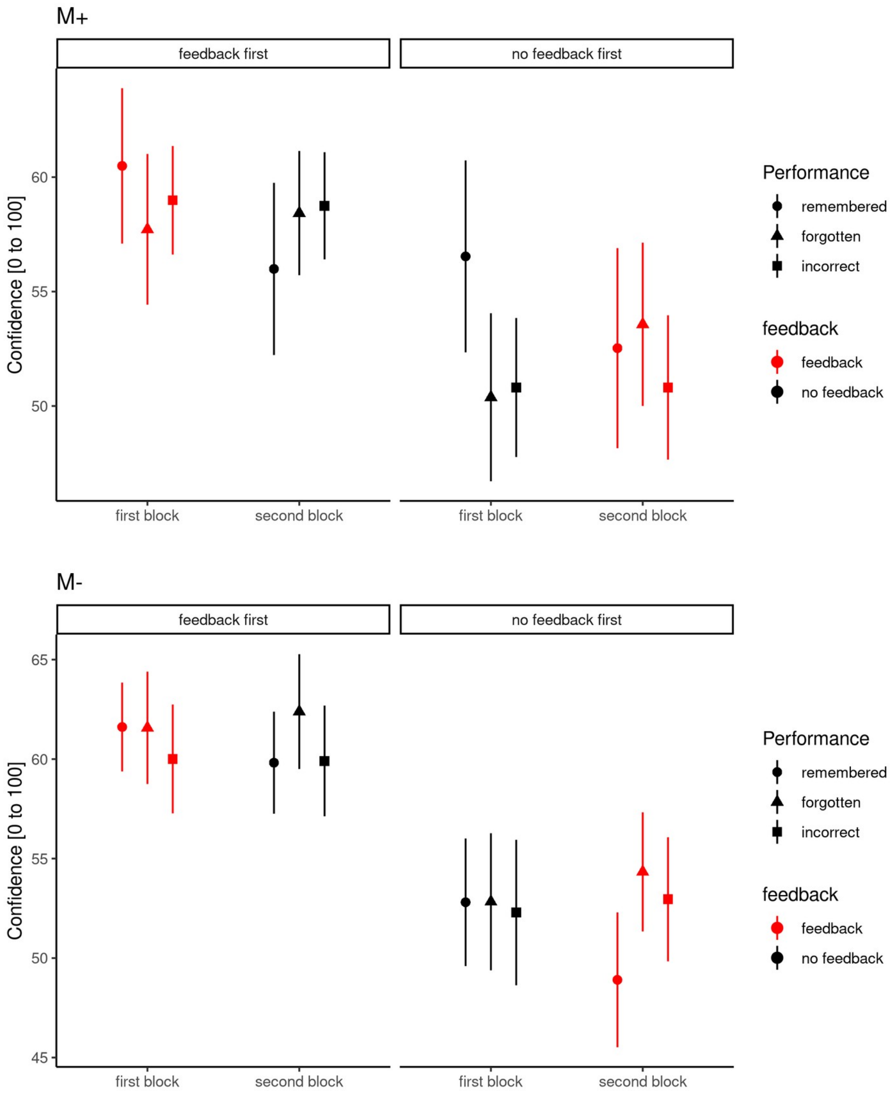

Supplementary Figure 14: Condition-specific group means and between-subject SEM for Confidence ratings on day 2 collected during the recognition test, sorted by performance on day 2 (remembered/forgotten/incorrect). Upper panel: M+ (dopamine-dependent trials). Lower panel: M- (dopamine independent trials). Left part of graphs: feedback first group; Right Part of graphs: no feedback first group.

Effect	df	Chisq	p
congruence	1	0.04	.838
feedback	1	0.05	.836
order	1	3.64 +	.057
RememForgot	2	0.83	.675
congruence:feedback	1	0.97	.310
congruence:order	1	032	.575
feedback:order	1	1.71	.200
congruence:RememForgot	2	1.78	.414
feedback:RememForgot	2	1.66	.449
order:RememForgot	2	1.15	.572
congruence:feedback:order	1	0.24	.626
congruence:feedback:RememForgot	2	2.53	.265
congruence:order:RememForgot	2	5.13 +	.076
feedback:order:RememForgot	2	3.53	.165
congruence:feedback:order:RememForgot	2	0.24	.901

Supplementary Table 14: Mixed ANOVA statistical results for confidence ratings on day 2 collected during the recognition test, based on performance on day 2 (remembered/forgotten). Mixed models were estimated with *congruence*, *feedback*, *feedback-order* and *rememForgot* as independent variables. Note that Factor "*rememForgot*" has 3 levels: Remembered (correct on both days), forgotten (correct only on day 1) or incorrect (already on day1 incorrect). A random intercept per subject was included. P-values were calculated with parametric bootstrapping. df = degrees of freedom; Chisq = chi square value; p = probability. "X:Y" denotes the interaction of X with Y.

For confidence ratings on day 2 (recognition test) showed a non-significant trend for order (p = .057) such that participants who started with feedback in the first half showed higher confidence than those who started without feedback. This effect was further qualified by a non-significant trend for a three-way interaction with *Feedback* and *RememForgot*. Post-hoc tests revealed that this effect was driven by the confidence ratings in the group who started without feedback: specifically, for remembered items in the no-feedback blocks ratings were higher than the confidence ratings for forgotten and incorrect trials in the same blocks, as well as higher as all ratings in the feedback blocks, but still lower than all ratings in the group who started with feedback. This effect was similar to the effects observed in experiment 1 where the triple interaction reached significance.

Discussion

Behavioral responses are often accompanied by particular reward-related emotional, motivational and metacognitive states. Previous studies on our new-word learning paradigm demonstrated that a modulation in SN/VTA was tightly linked to enhanced pleasure and confidence ratings, whereas arousal ratings were not enhanced (Ripolles, 2016, 2018). To relateour findings to the previous ones more closely, we also measured trial-based arousal and pleasantness ratings on day 1 in addition to performance. Replicating previous findings, for pleasure ratings we again found a larger difference for correct relative to incorrect responses in the no-feedback M+ conditions than the no-feedback M- conditions (see Supplementary Materials 1,3). No such differences between no-feedback M+ and M- conditions were observed for the arousal ratings in the tightly controlled laboratory settings again replicating previous observations (see Supplementary Materials 2), though there was some effect in the less controlled online experiment. Moreover, we also observed an effect of feedback on pleasantness ratings following correct vs. incorrect responses, with larger differences for feedback trials (see Supplementary Materials 4). Please note that ratings were obtained after feedback, hence this rating might have interacted with feedback in addition to intrinsic signaling.

In previous studies we had never assessed subjective states during the recognition period. Here, adding to previous observations, we also assessed subjective states on day 2; in particular arousal, pleasantness plus confidence (note that confidence was not included on day 1 since ratings were acquired after feedback; no feedback was provided on day 2 rendering the confidence judgements more meaningful). For pleasantness, we observed higher ratings for no-feedback M+ trials than feedback M+ trials and opposite effects for M- trials suggesting that external feedback on day 1 had interfered with intrinsic dopaminergic signaling in the laboratory experiment (Supplementary Figure 5). Moreover, we observed higher pleasantness ratings for remembered M+ trials relative to incorrect and forgotten M+ trials; and opposite effects for M- trials suggesting enhanced dopaminergic signaling during correct remembering which may indicate additional dopaminergic reconsolidation processes (Redondo et al., 2011). Arousal ratings did not show any differences on day 2 (Supplementary Figure 6). In the online experiment, no significant effects were observed despite the use of a more fine-grained scale.

Finally, confidence ratings, which may reflect complementary metacognitive processing, were most enhanced for remembered M+ words relative to forgotten and incorrect M+ words in the no feedback first group. This pattern was replicated as a trend in the online version of the experiment.
